# Time-Resolved Killing of Individual Bacterial Cells by a Polycationic Antimicrobial Polymer

**DOI:** 10.1021/acsbiomaterials.4c00263

**Published:** 2024-03-29

**Authors:** Zachary Benmamoun, Prem Chandar, Joe Jankolovits, William A. Ducker

**Affiliations:** † Department of Chemical Engineering, Virginia Tech, Blacksburg, Virginia 24060, United States; ‡ Unilever Research, Trumbull, Connecticut 06611, United States

**Keywords:** cationic polymer, adsorption, bacteria, antimicrobial, PDADMAC, *E. coli*

## Abstract

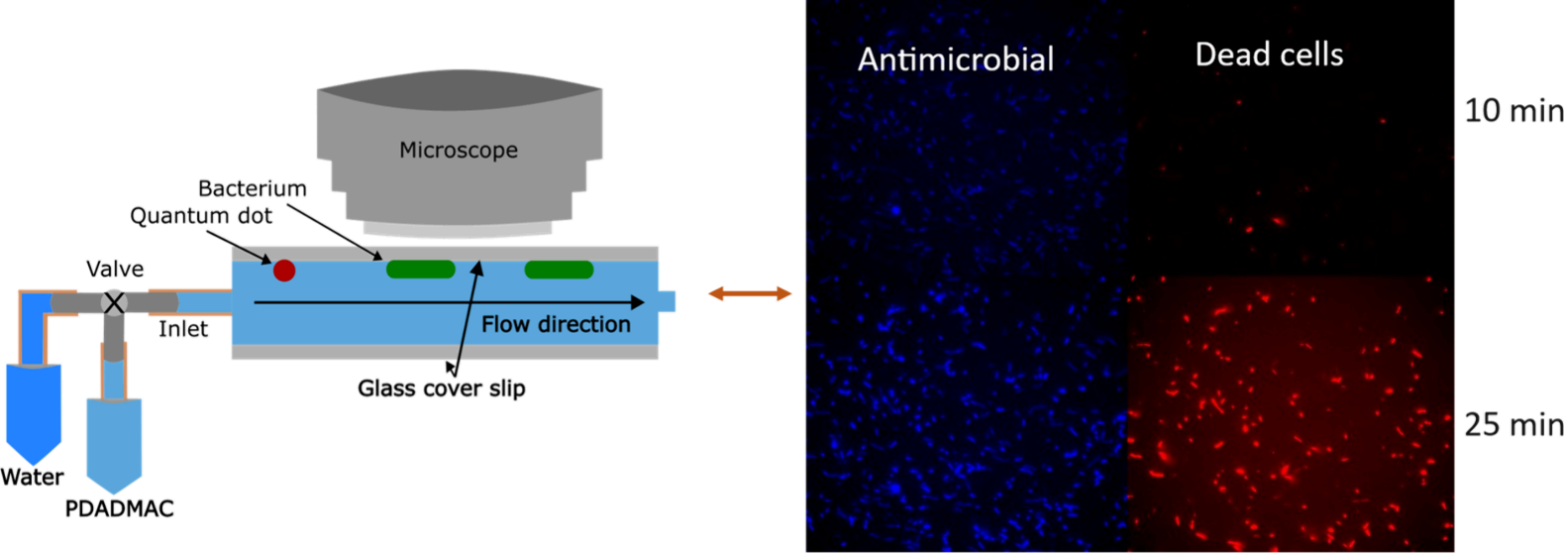

Polycationic polymers are widely studied antiseptics, and their efficacy is usually quantified by the solution concentration required to kill a fraction of a population of cells (e.g., by Minimum Bactericidal Concentration (MBC)). Here we describe how the response to a polycationic antimicrobial varies greatly among members of even a monoclonal population of bacteria bathed in a single common antimicrobial concentration. We use fluorescence microscopy to measure the adsorption of a labeled cationic polymer, polydiallyldimethylammmonium chloride (PDADMAC, *M*_w_ ≈ 4 × 10^5^ g mol^–1^) and the time course of cell response via a cell permeability indicator for each member of an ensemble of either *Escherichia coli, Staphylococcus aureus*, or *Pseudomonas aeruginosa* cells. This is a departure from traditional methods of evaluating synthetic antimicrobials, which typically measure the overall response of a collection of cells at a particular time and therefore do not assess the diversity within a population. Cells typically die after they reach a threshold adsorption of PDADMAC, but not always. There is a substantial time lag of about 5–10 min between adsorption and death, and the time to die of an individual cell is well correlated with the rate of adsorption. The amount adsorbed and the time-to-die differ among species but follow a trend of more adsorption on more negatively charged species, as expected for a cationic polymer. The study of individual cells via time-lapse microscopy reveals additional details that are lost when measuring ensemble properties at a particular time.

## Introduction

Polycations are positively charged polymeric materials that often display antimicrobial properties^1−4^ and are utilized in the food industry and water treatment^5^ and in the design of antimicrobial surfaces.^6,7^ While research on polycations is widespread, the mechanisms of antimicrobial action are not fully understood, and an improved understanding could lead to improved performance. Various mechanisms have been proposed, which all begin with adsorption of polycation onto the cell surface,^8−14^ followed by an increase in membrane permeability, which leads to death.^15^ Several works have confirmed that adsorption does occur.^2,16,17^

Although the mechanism of killing is thought to be adsorption to the cell membrane followed by lysis, there are few papers that describe the quantitative relationship between the adsorbed amount and cell death. Quantitation of antimicrobial activity continues to be in terms of the solution concentration of antimicrobial required to kill or inhibit growth using metrics such as Minimum Inhibitory Concentration (MIC) and Minimum Bactericidal Concentration (MBC).^17,18^ Here we examine the hypothesis that cell death occurs after the antimicrobial reaches a threshold density on the cell surface rather than at a critical solution concentration. Therefore, our interest is in the adsorption required for cell death and the time to die after adsorption.

Most studies and metrics of antimicrobials measure effects on the entire population (e.g., MIC), whereas evidence shows a diversity of effects across populations, e.g.,^19−21^ including antibiotic resistance.^22−26^ Therefore, we examined the polymer adsorption for each individual cell in a population to assess the diversity of the response. This follows past work where adsorption of (cationic) peptide antimicrobials was studied for individual *Escherichia coli* (*E. coli*) cells.^17,27^

In this work, we studied the effect of the synthetic cationic polymer polydiallyldimethylammmonium chloride (PDADMAC) on individual cells of *Escherichia coli* (*E. coli*), *Staphylococcus aureus* (*S. aureus*), and *Pseudomonas aeruginosa* (*P. aeruginosa*) in water without growth medium or added electrolyte in order to simulate fluids outside the body, where synthetic antimicrobial polymers can be deployed. These bacteria are representatives of each of the two major subgroups of bacteria: gram-negative and gram-positive. The cells are not growing or dividing under the conditions studied.

PDADMAC is a cationic polymer, which is an important class of antimicrobial,^28^ and is an effective antimicrobial in bulk solution,^29^ and at surfaces.^30−34^*E. coli* is frequently implicated in foodborne and waterborne infections, S. *aureus* is commonly transmitted on surfaces, and *P. aeruginosa* is commonly transmitted through aerosols or through surfaces. For fast measurement on an individual cell basis, we use time-lapse fluorescence microscopy of live bacteria in a flow cell that allows us to simultaneously track all the cells in a field of view (100–200) over time. To enable measurement of adsorption, we labeled the PDADMAC with the fluorescent dye, Cy3. Cell response was measured with a “dead stain”, SYTOX Blue, which becomes fluorescent when the cell membrane becomes permeable, and SYTOX Blue is able to bind to DNA.

Traditional methods of evaluating antimicrobial efficacy report the concentration of antimicrobial required to kill the cell,^6,35,3,34^ but we find that death is determined by the time since the cells were exposed to a threshold density of adsorption. The density of adsorption depends on the concentration in solution, but the two measures are not the same if the cell surface varies across the population. We find that the adsorption of the antimicrobial varies greatly from cell to cell at the same solution concentration and time, which implies that there is a distribution of cell surface properties. Adsorption takes some time; therefore, exposure to the same solution concentration leads to more adsorption over time. We show that the polymer solution concentration is not the sole determinant for death, as the amount of PDADMAC required to kill a cell remains the same for different solution concentrations. This suggests that common measures, such as MIC and MBC, obscure much of the richness that occurs in antimicrobial treatments. We find that surviving cells have, on average, less adsorbed PDADMAC than did killed cells. Finally, we find that *S. aureus* dies faster than *E. coli* and *P. aeruginosa*, even though PDADMAC adsorbs most densely on *E. coli*, and least densely on *P. aeruginosa*. We emphasize the importance of studying the distribution of cell behaviors rather than the average behavior.

## Materials and Methods

### Materials

Cy3-labeled polydiallyldimethylammmonium chloride (Cy3-PDADMAC) with a labeling degree of 1:196 was purchased from Surflay Nanotec (Berlin, Germany). The polymer was synthesized by copolymerization of the labeled and unlabeled monometers. Size exclusion chromatography of the tagged polymer showed a bimodal molecular weight distribution with a number-average molecular wight of 3.0 × 10^5^ g/mol for one mode and 4.6 × 10^5^ for the other mode (Figure S1). So that variation in polymer labeling is smoothed out across a cell, it is better if many polymers adsorb to each cell. Assuming the polymer lies flat (i.e., giving the lower bound for number of molecules) and assuming a monolayer coverage, there are about 3000 polymer molecules per cell, which should be enough to average out variation in labeling. SYTOX Blue Dead Cell Stain was purchased from Fischer Scientific. CdSe/ZnS core–shell quantum dots stabilized with octadecylamine ligands were purchased from Millipore Sigma.

### Growth of Microbial Strains

*E. coli*, *S. aureus*, and *P. aeruginosa* were selected as species where pathogenic strains cause widespread illness, and the ATCC 25922, ATCC 6538, and PAO1 strains for *E. coli*, *S. aureus*, and *P. aeruginosa* respectively were selected because of their widespread use as standards for antimicrobial testing.^36,37^ Frozen stock was streaked onto a plate and incubated for 24 h at 37 °C. Once grown, a single colony was selected so that we began with cells that were almost monoclonal. Cells from that colony were grown in 10 mL of Tryptic Soy Broth (TSB, BD, Sparks, MD) to stationary phase for 48 h at 37 °C with aeration (100 rpm). Following growth, cell purity was verified by streaking onto Tryptic Soy Agar (TSA, BD) and incubating at 37 °C for 24 h. If cell purity was confirmed, the cell suspension grown earlier for 48 h was used for experiments. For CFU experiments, cell density was diluted to 3 × 10^8^ CFU/mL and resuspended in deionized water or a buffer solution. For injection into the flow cell, the cell suspension was directly used.

### Flow Cell

Parallel-plate flow chambers were constructed from polycarbonate (Figure S2). The inlet and outlet were 1 mm in diameter. The channel of the flow cell (*L* × *W* × *H*) was 40 × 2 × 1 mm^3^. Video microscopy occurred midway between the inlet and outlet. Cover glass (no. 1, 25 mm × 50 mm^3^, Fischer Scientific) was used as the flow cell windows. To serve as an intensity standard, quantum dots (QDs) were deposited onto the interior surface for the flow cell by drop casting an ethanol suspension onto the coverslip prior to assembly of the flow cell. Bacterial suspension was injected into the flow cell with a syringe, then the flow cell was inverted and allowed to sit for 20 min to encourage bacteria to contact the coverslip window. After 20 min, TSB was flowed through the flow cell at 4 mL/h for 20 min to remove unadhered cells. Once unadhered cells were removed, a Cy3-PDADMAC and SYTOX Blue solution were flowed over the cells at a volumetric flow rate of 4 mL/h. Preliminary experiments with rhodamine 6G demonstrated that the dye reached the imaging point less than 30 s after the dye was added. This time period was small compared to that of other time scales in the experiment. SYTOX Blue was used to measure the viability of the cells, and the cells flowed continuously for the duration of the experiment. When SYTOX Blue is injected into the flow cell, there is immediately a small amount of fluorescence for a few seconds, which we attribute to binding of extracellular DNA, so we do not include this initial fluorescence in our analysis. A control experiment shows that cells that are rinsed with propidium iodide immediately prior to exposure to SYTOX Blue exhibit fluorescence in the propidium iodide channel but not in the SYTOX Blue channel, presumably because the extracellular DNA has bound to propidium iodide and washed away. Another preliminary experiment using cells that were killed with ethanol showed that SYTOX Blue enters the cell and fluoresces almost instantly after arrival (Figure S3). We measured the viability of cells in water–SYTOX Blue solution and found that cells died after ∼ 100 min (Figure S4), so data collected past that time was not considered further. The base solution was water rather than buffer, because antimicrobials are used outside the body, where liquids are usually more similar to water than serum.

### Imaging

Time-lapse photographs with a period of 30 s were obtained by using a Zeiss Imager. M2 fluorescence microscope with a 63× objective. Three images were obtained at each time point, a phase contrast channel to view the cells and determine cell size and eccentricity, a fluorescence channel (10 ms exposure time) to view the labeled polymer (PDADMAC) and a second fluorescence channel (20 ms exposure time) to image the SYTOX Blue stain. All color in the images is false color. These two fluorophores have widely separated adsorption/emission so there is little interference of the spectra, and the exposures are very short compared to the period between images so the images of bacteria, polymer, and dead stain are effectively simultaneous. Although SYTOX Blue emits a blue color, we artificially assigned a red color to these images for consistency with the reader experience of dead stains (e.g., propidium iodide) being shown in red. These images were analyzed using Trackpy (https://github.com/soft-matter/trackpy), an ID and tracking software, as well as custom code.

Quantitative data was obtained from videos using Python software. The time-lapse images were saved as.tif files, then imported into Python using skimage.io, then thresholded (see Figure S5 for an excerpt of the Python script) using Otsu thresholding. The thresholding was done to subtract the background signal to improve cell detection. Trackpy Python script (https://github.com/soft-matter/trackpy) was used to identify and track cells in greyscale images and then to obtain brightness, size, particle number, location, and eccentricity of each cell. Cells in the PDADMAC, SYTOX Blue, and phase contrast channels were matched using their coordinates and assigned a particle number. Cell brightness (signal level for each pixel) was divided by the square of the cell size to obtain intensity. Intensity from replicate experiments and for different conditions was transformed to normalized intensity by comparison to the average quantum dot intensity in the same field of view at the same time (See Figure S6 for further details). Cells that were dead at the start of the experiment (stained with SYTOX Blue at the start) were not included in data analysis. The Supporting Information contains a calculation suggesting that owing to the large number of monomers adsorbed, the variation in degree of labeling will not strongly affect the results.

### Measurement of Cell Number

The number of bacterial cells in a suspension was measured as colony-forming units per milliliter of suspension (CFU/mL). This measures the number of viable cells, i.e., those cells that were able to grow into a colony. A 10 μL droplet of bacterial cell suspension was mixed with 90 μL of an antimicrobial solution. A 10-fold dilution series was prepared for each cell suspension; 0.1 mL of each dilution was spread on TSA in triplicate, and colonies were counted after 24 h of incubation at 37 °C. If no colonies were present for the least dilution, we rounded the result up to one colony to enable a log transformation. We set this as the detection limit shown in the figures.

### Statistics

In general, we found that distributions of cell behavior were usually highly right-skewed, so the assumption of normality was not justified for the population distributions. Our response was to show the entire distribution and, when hypothesis tests were needed, to use the nonparametric Mann–Whitney U test that compares the ranking of scores when two conditions are combined, which is similar to a comparison of medians. In some cases, the means of distributions were compared by invoking the central limit theorem to allow the Student’s *t* test. Where Box and Whisker plots are shown, the green dotted line represents the mean, and the orange solid line represents the median, which is lower in almost each case. In general, we used about 95% confidence for the significance.

## Results and Discussion

### Cells are Sensitive to PDADMAC Concentrations of about 10 μg/mL

We determined the appropriate PDADMAC concentration to kill *E. coli* in our flow cell measurements by using the traditional colony forming unit (CFU) counting technique (Figure S7). The CFU technique counts cells that can divide and grow enough times to form a visible bacterial colony. About 99% of suspended *E. coli* cells died (2-log kill) after 5 min of exposure to 10 μg/mL PDADMAC so flow cell experiments focus on concentrations near 10 μg/mL. Note that a typical flow cell experiment contained hundreds of cells, so only 0–10 live cells were expected in a field of view after exposure to PDADMAC.

### Cells Die about 5–10 min after PDADMAC Adsorbs

The mechanism of polycation antibacterial activity is in the process of being understood, but it seems reasonable that the polycation must adsorb rather than act from a distance. Our first goal was to verify that adsorption is essential for cell death. As described earlier, our measurement of cell death was actually a measure of cell wall permeability, but for simplicity, we will describe emission from the SYTOX Blue as if it indicated cell “death”. About 1/200 of the monomers of PDADMAC were labeled with the fluorescent dye, Cy3, to enable quantification of adsorption, but from here we will simply refer to the tagged polymer as PDADMAC.

**Figure 1:**
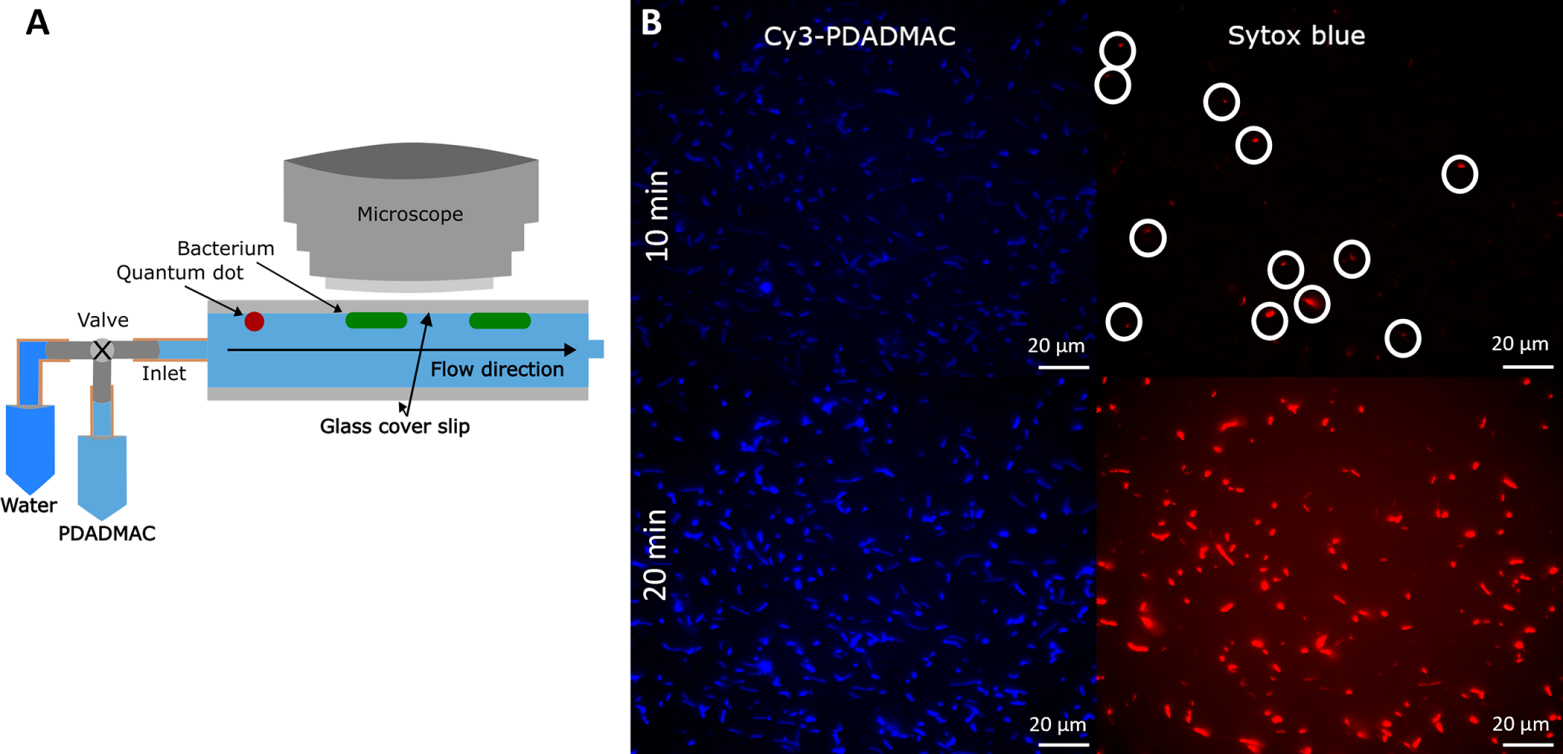
(A) Schematic of flow cell experiment. PDADMAC flow was followed by water flow; the water was used to limit the exposure time to PDADMAC in solution. (B) Fluorescence microscopy of adhered *E. coli* cells 10 and 20 min after 10 μg/mL Cy3-PDADMAC was flowed over the cells. Two channels are shown: Cy3 fluorescence for identifying polymer adsorption (blue color) and SYTOX Blue fluorescence to identify the timing of permeation of the cell membrane, i.e., cell death (red color). Some cells were dead at the start of the experiment, as indicated by the white circles.

Flow cell experiments (Figure 1A) showed that there was a time-lag between adsorption and death. About 10 min after PDADMAC adsorbed, new dead cells are observed and there is a low density of polymer on other cells (faint blue color in Figure 1B), but they are not yet dead as indicated by the lack of red-stained bacteria in Figure 1. After 30 min there is a much greater density of polymer on cells and many more dead cells.

**Figure 2:**
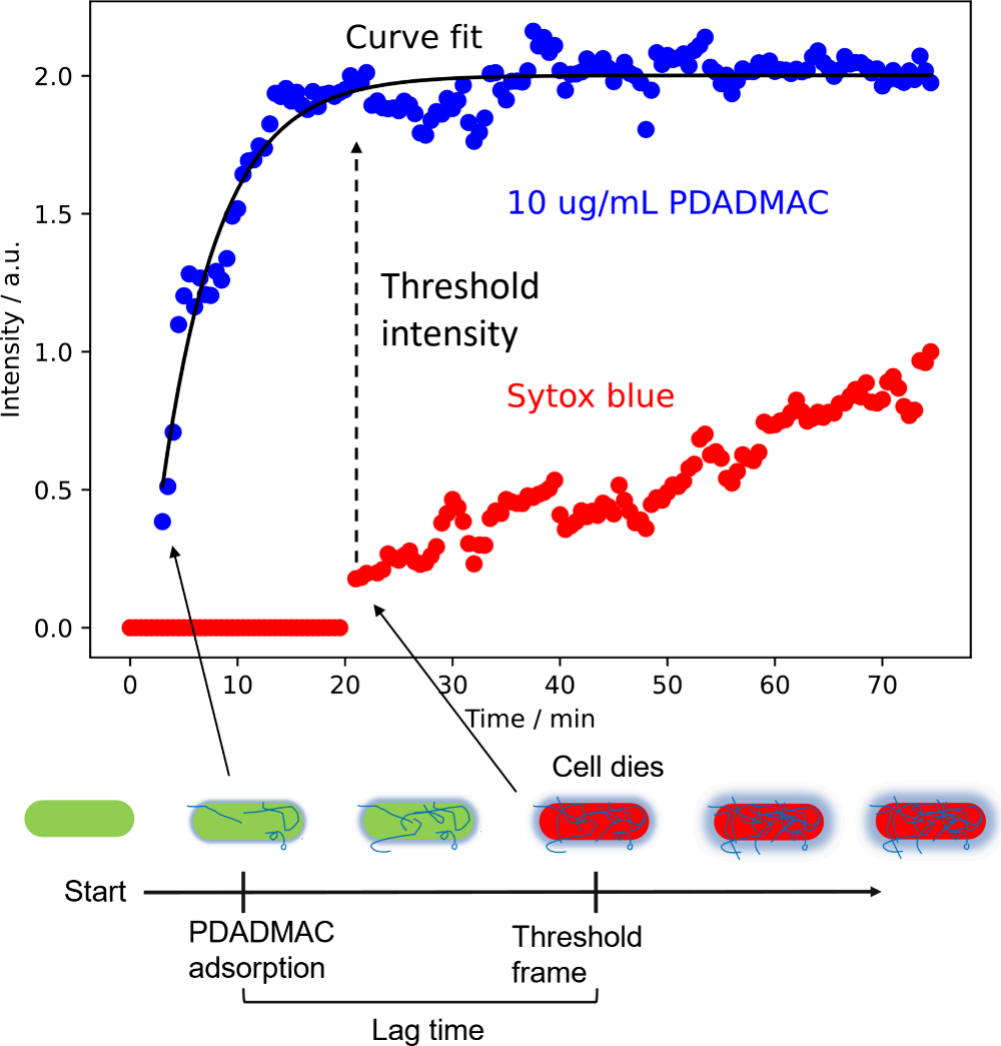
Time-course of adsorption and permeation of a single *E. coli* cell. (A) Time-course of fluorescence emission when 10 μg/mL PDADMAC polymer was flowed over the cells. There is a rapid rise in PDADMAC adsorption in the period 5∼15 min (blue symbols). The SYTOX Blue intensity increases rapidly from zero intensity at about ∼20 min, indicating cell permeation (death). Sometimes there is a slow decline in signal intensity late in the experiment, which we attribute to photobleaching. The black line indicates the fit to eq 1. (B) Schematic showing the adsorption of polymer to the cell and the definition of lag time and threshold frame. The lag time is the time between the first adsorption of the polymer (as determined by the value of *t*_0_ obtained from the fit to eq 1) and an increase in the SYTOX Blue intensity (see arrows).

For quantitative conclusions, videos were analyzed using software that identified each bacterium and tracked each one over the course of the experiment. The fluorescence emissions from both the dead stain and the polymer were recorded for each cell in each frame. Figure 2A shows an example of a time-course of emission for one cell, demonstrating that adsorption takes some time and that there is a lag between adsorption and cell death. Zero time indicates when PDADMAC was injected into the flow cell. Within 30 s, the flow window becomes fluorescent, indicating that the PDADMAC has reached the cells. Within 1–2 min, cells become fluorescent, indicating adsorption to the cells. Adsorption increases rapidly over the following 5 min. Cell death was judged to occur when the dead stain fluorescence signal increased for an individual cell. After the flow solution was switched to water, the polymer remained adsorbed, indicating that the adsorption was irreversible. Figure 2B is a schematic showing our interpretation of the data and the nomenclature used. The lag-time is defined as the difference between an increase in SYTOX Blue intensity and the onset of PDADMAC adsorption. In a control experiment, where dead but intact cells were exposed to the dead stain, the dead stain became fluorescent within seconds of arrival at the cell surface, indicating that the time lag was not due to the transport time of the dead stain through the cytoplasm but is instead the time required for the cell membrane to become permeable (Figure S3). We additionally find that cells killed by PDADMAC retain their overall structure as observed by light microscopy (Figure S8); the damage to cells is nanoscopic, it does not cause loss of the micrometer-level structure. We therefore conclude that adsorption to cells occurs quickly but permeabilization of the cell membrane is a slow process that takes minutes.

From Figure 2A the adsorption of PDADMAC occurs over about 10–15 min. To simplify comparison among members of a population of cells, we reduced this time-course to three parameters using a fit of the fluorescence emission, *N*, to the half-logistic equation:
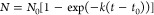
1where *N*_0_ is the saturation absorption, *t* is time, *t*_0_ is the first time at which the intensity exceeds the threshold, and *k* is a fitting parameter that describes the rate of increase. The justification for using this equation is that it provides a reasonable fit to the data, but it can be derived, as shown in the Supporting Information, assuming that there is a fixed number of available sites for polymer adsorption at any given solution condition. This assumption is clearly an approximation for a charged polymer adsorbing to a charged bacterial cell, but the important point is that the equation allows us to summarize the time-course through the parameter, *k*, and determine the time of initial adsorption, *t*_0_, which is used in calculating the lag time.

**Figure 3:**
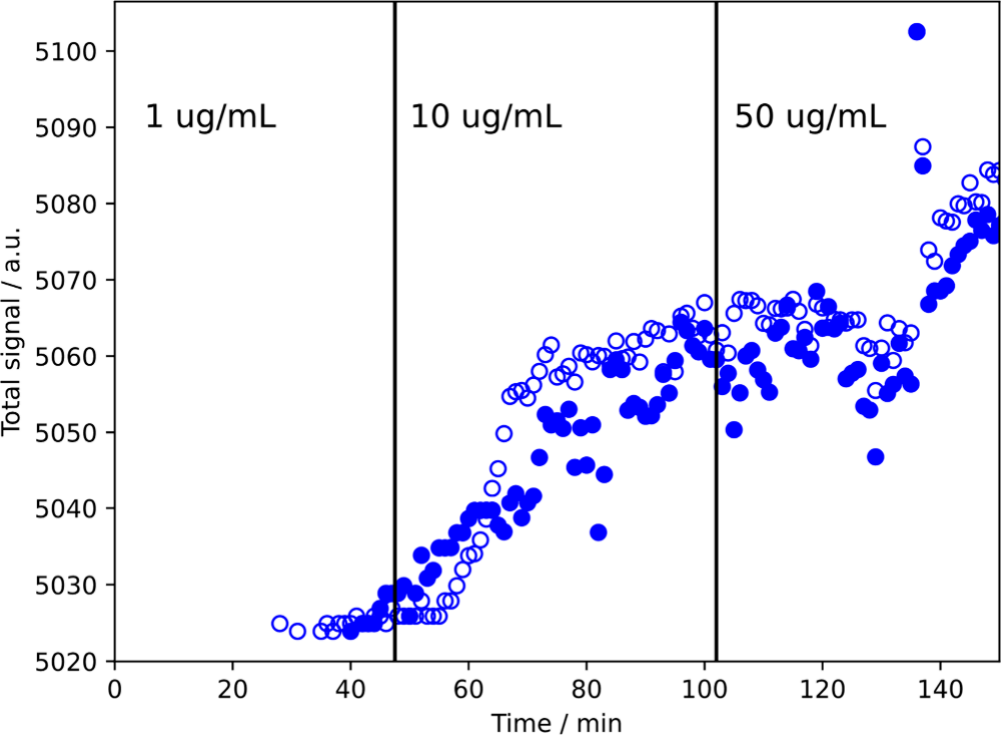
Time course of adsorption of PDADMAC for two *E. coli* cells when the solution concentration was increased three times. The three concentrations are 1 μg/mL, 10 μg/mL, and 50 μg/mL and the periods at a constant concentration are separated by vertical black lines (the 1 μg/mL begins at time = 0) The adsorption increased each time the concentration was increased.

Traditional MIC experiments observe better killing at higher concentrations of antimicrobials, so for consistency we would expect more adsorption at higher concentrations. This is also expected from mass action up to the point of saturation of the surface. We tested this by measuring the polymer intensity as the PDADMAC concentration was increased stepwise from 1 μg/mL to 50 μg/mL, as shown in Figure 3. More polymer clearly adsorbs at higher concentrations.

One potential issue with using emission as a quantitative measure is that the intensity can vary with the intensity of the source light, the microscope focus, etc. To better quantify the PDADMAC data, we used fluorescent quantum dots (QD) as internal intensity calibration standards. The QDs were irreversibly adsorbed on the interior surface of the flow cell window, so that they were in the same focal plane at the same time as the polymer. Total PDADMAC signal for the cell was normalized by the square of cell size to obtain intensity and that intensity was normalized by the average quantum dot intensity (Figure S6). All fluorescence data described here are normalized intensity.

### There is Great Diversity in Cell Response

**Figure 4:**
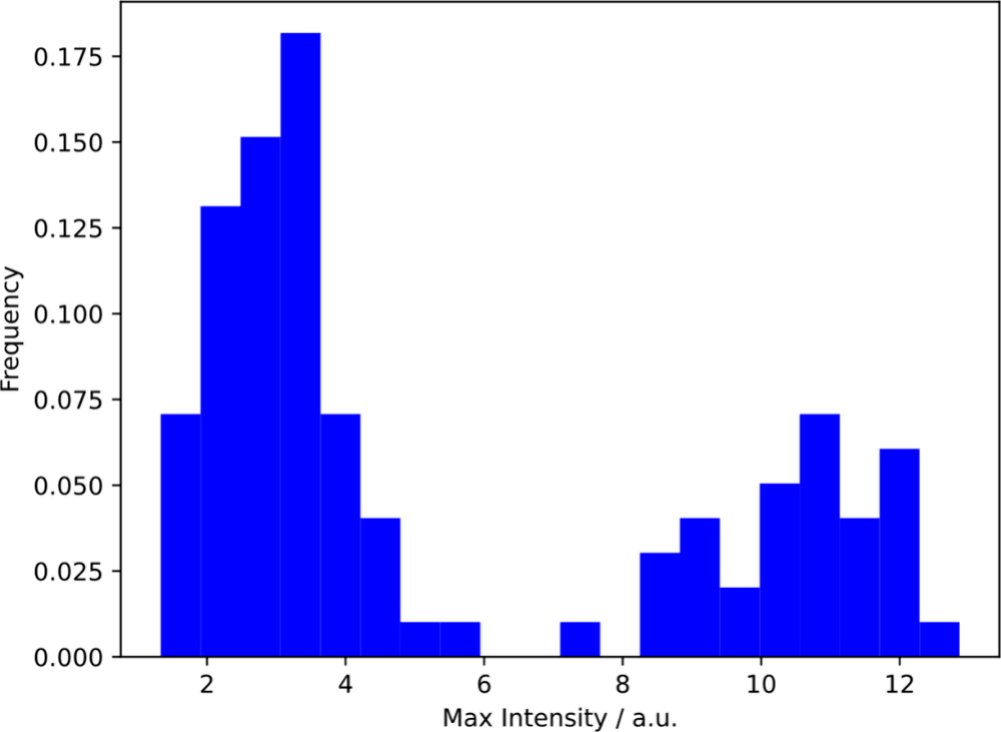
Maximum intensity of *E. coli* cells after exposure to 10 μg/mL PDADMAC. Data are for a single experimental run. There is considerable diversity of response to PDADMAC for an essentially monoclonal sample.

We observed a very broad distribution of PDADMAC adsorption characteristics onto cells. Figure 4 shows a histogram of the maximum PDADMAC fluorescence intensities for each cell in a single experiment. Clearly there is a huge range of adsorption (a factor of 12), even for the monoclonal population that we study. PDADMAC is cationic, and the adsorption is likely dominated by charge–charge interactions. If so, then other cations should also show a range of adsorption. Figure S9 demonstrates that Rhodamine 6G, a cationic fluorophore also shows a large range of adsorption densities on *E. coli* cells. Prior work has shown that polycations typically adsorb with about 1–2× as many charged groups as were originally present on the surface.^38^ The vast range in the adsorption of PDADMAC suggests that there was a very broad range in surface charge of the bacteria prior to adsorption of the polymer. If the bacteria behave similarly to previously measured work on silica and polystyrene lattices, then the bacteria with an adsorbed polymer will be positively charged after adsorption.

The rate of adsorption also varies among the cells. Figure S10 plots the lag time vs the rate of polymer adsorption (fitted *k* using eq 1). First note that the lag time varies enormously. The adsorption rate has a bimodal distribution, and cells that adsorb PDADMAC faster tend to die faster.

This result raises the question of whether the observed diversity is a property of the cells or is produced by inhomogeneous mixing in the flow cell. An experiment where cells in suspension were mixed with PDADMAC then imaged showed a similar diversity of fluorescence (Figure S11), demonstrating that the diversity is a property of the *E. coli*. We conclude that there is considerable heterogeneity in the cell response to antimicrobials, and this is one of the main themes of this manuscript.

### There is a Characteristic Adsorbed Amount at Death

**Figure 5:**
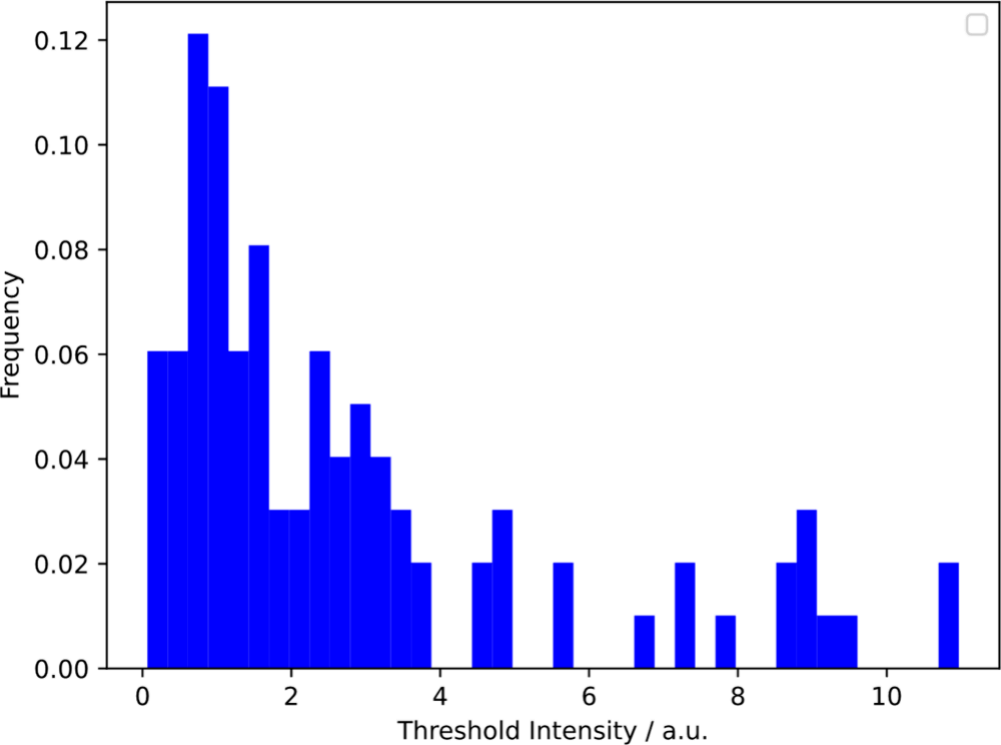
PDADMAC adsorption at the time of *E. coli* cell death for 10 μg/mL bulk PDADMAC. Note that most cells die with an intensity between about 0.5 and 4 but there is considerable diversity. Mean = 2.8; standard deviation = 2.7.

We hypothesized that cell death would occur after a sufficient density of polymer was adsorbed on the cell. Despite the broad diversity of cell response, we do find that there is a characteristic adsorbed amount at the time of death. As shown in Figure 5, many cells die when the intensity of emission is about 1. Clearly some cells have much more polymer adsorbed at the time of death.

### There is a Time Lag between Adsorption and Cell Death

**Figure 6:**
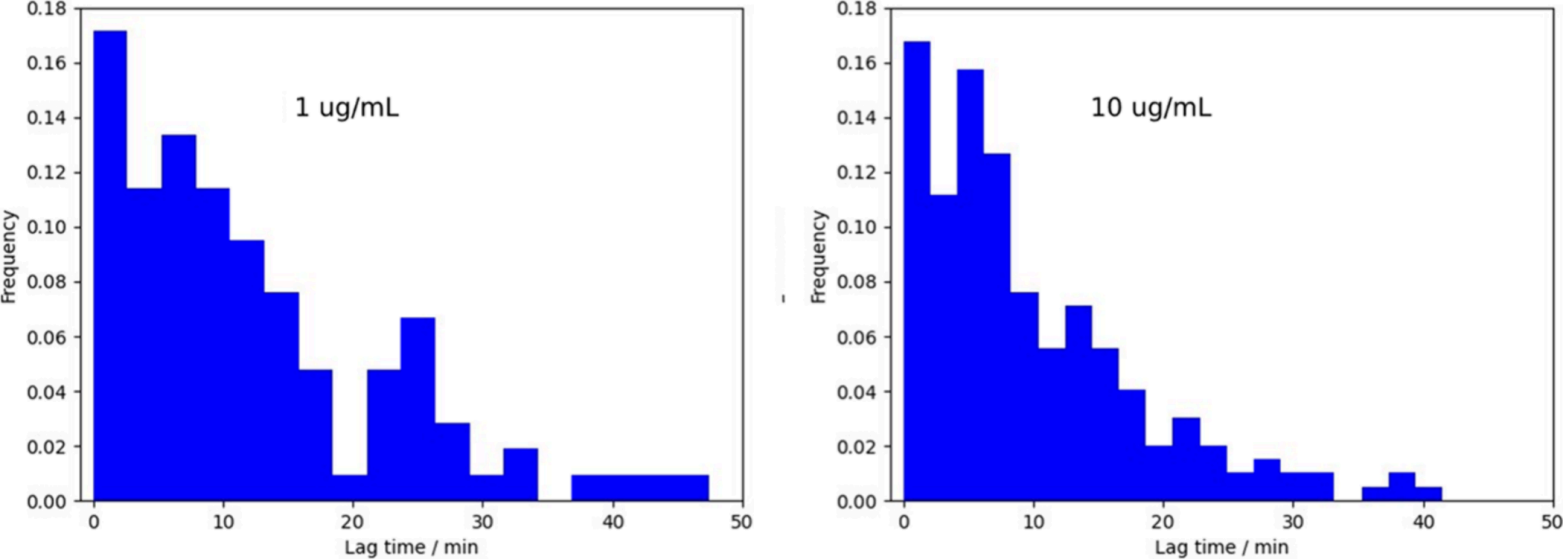
Histogram of time lag between initial PDADMAC adsorption and *E. coli* cell death for 1 and 10 μg/mL PDADMAC flow experiments. See Figure 2B for nomenclature. 1 μg/mL mean = 13 min; standard deviation = 12 min; 10 μg/mL mean = 9.8 min; standard deviation = 8.5 min. There is considerable diversity of cell response, and a lower PDADMAC concentration led to a longer lag between adsorption and death.

Figure 2 shows one example of the time lag between PDADMAC adsorption and cell death. To quantify this lag, we consider the initial adsorption, which we obtain from the fit of *t*_0_ from eq 1. This is more precise than relying on the first nonzero point. The histogram of lag times (Figure 6) shows that there is very noticeable lag between adsorption and death (about 10 min) and that the lag time decreases with increasing PDADMAC concentration.

As shown in Figure 6, a mean lag time of *∼* 10 min was characteristic for 10 μg/mL PDADMAC exposure *to E. coli* and this lag time increased to 13 min when only 1 μg/mL PDADMAC concentration was available.

### The Lag Time Leads to an Overestimate of the Adsorption Density for Cell Death

The lag time between adsorption and death complicates the interpretation of the adsorption required for death. The density of PDADMAC adsorption can continue to increase during the lag time, and therefore, adsorption may increase after the cell has already received a lethal dose. If this is true, then the measurement of the PDADMAC adsorption at death is an overestimate of the lethal dose. For example, during the lag period in Figure 2A, 1.3 units of PDADMAC are adsorbed for 7 min and an additional 1.7 units for the final 2 min.

To determine the minimum dose for death, we performed switch-flow experiments, where the flow of PDADMAC was stopped and replaced with a flow of water. The experiment was repeated at the same PDADMAC concentration but with a shorter time of exposure to the PDADMAC solution. Recall that there is little desorption in water after PDADMAC solution is switched for water (Figure 2 and Figure S12).

**Figure 7:**
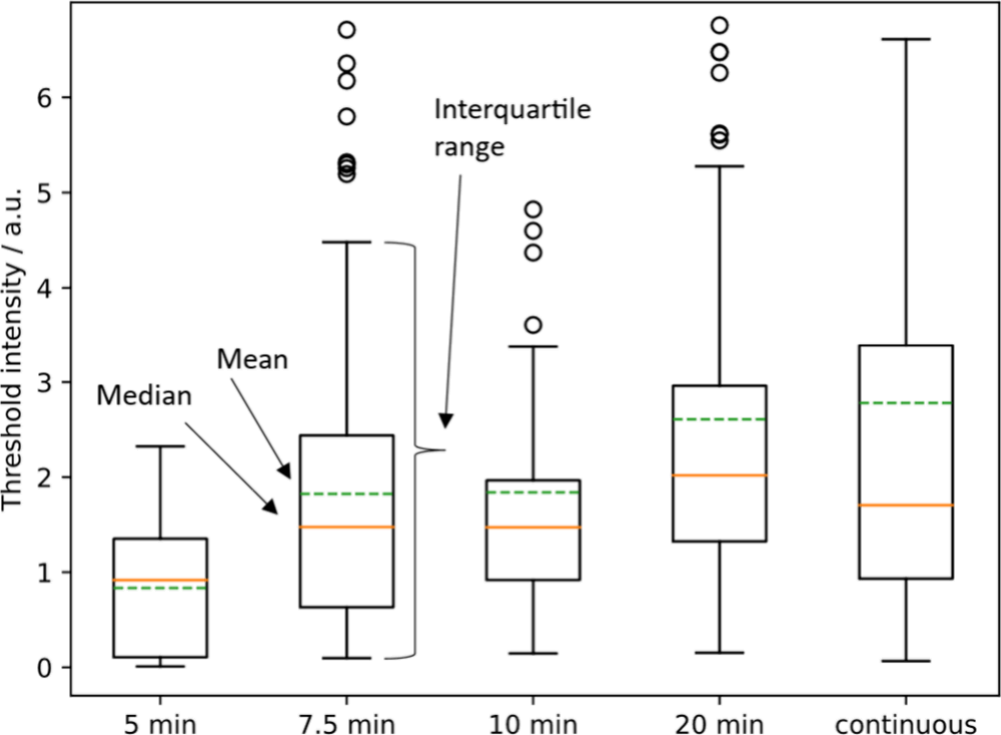
Threshold intensities for 10 μg/mL PDADMAC onto *E. coli* in switch flow experiments. The whiskers are Q1 – (1.5 × IQR) (truncated at zero) or Q3 + (1.5 × IQR), where IQR is the Interquartile range. Beyond the whiskers, the data are considered an outlier, indicated by the hollow circles. There are about 100 data points for each time. Linear regression for the means of three replicates at each time in the period 5 to 20 min showed a positive slope (*p* = 0.045) demonstrating a lower threshold at shorter times. The threshold intensity is about 0.8 at 5 min. This data show that continuous flow experiments overestimate the lethal dose of PDADMAC.

The results of switch-flow experiments (Figure S13) for 10 and 20 min of PDADMAC flow showed that longer PDADMAC exposure times cause more adsorption. To determine the threshold PDADMAC adsorption for death, we performed a series of experiments with diminishing PDADMAC flow time until the threshold intensity remained constant. As shown in Figure 7, the minimum threshold intensity was reached at 5 min of PDADMAC flow, corresponding to an intensity of about 0.8. This is much lower than the density of adsorption that is recorded in the continuous flow experiment, which is about 2.8 on average. Therefore, continuous flow experiments overestimate the density of PDADMAC required to kill *E. coli*.

### The Threshold Adsorption is Independent of PDADMAC Concentration

**Figure 8:**
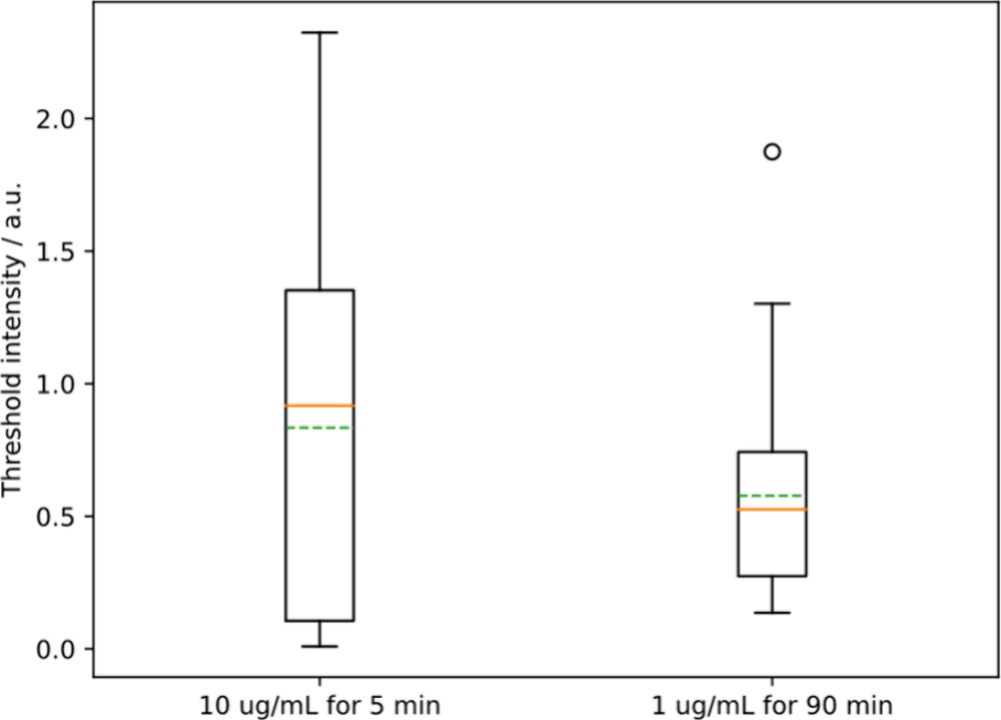
Threshold intensities for 1 and 10 μg/mL Cy3-PDADMAC in *E. coli* switch flow experiments. Minimum threshold intensity for 1 μg/mL switch flow experiment is the same as 10 μg/mL. Some outlier points do not appear on the graph. Mann–Whitney U test of medians, *p* = 0.3. *t* test between replicate means, *p* = 0.44. We interpret this to mean that the density of adsorption required for kill is not significantly different for different bulk concentrations of the polymer. A lower concentration of polymer does take a longer time to kill.

Our hypothesis was that cell death follows from a critical level of adsorption, not at a critical concentration. To test this, we compared the threshold adsorption for death in 1 and 10 μg/mL in switch flow experiments. Recall that the plateau adsorption densities are very different for these two concentrations (Figure 3). As can be seen in Figure 8, the minimum threshold intensity for PDADMAC is the same in 1 μg/mL as in 10 μg/mL (*t* test for means of three replicates, *p* = 0.44) Therefore, polymer surface density is more indicative of kill than solution concentration. This is a primary conclusion of the manuscript. Note that the times for the two concentrations are different because the lag time decreases with the concentration of PDADMAC: we needed to wait longer at 1 μg/mL concentration for the cells to die.

### Cells Takes Longer to Die with a Lower Surface Density of Antimicrobial

**Figure 9:**
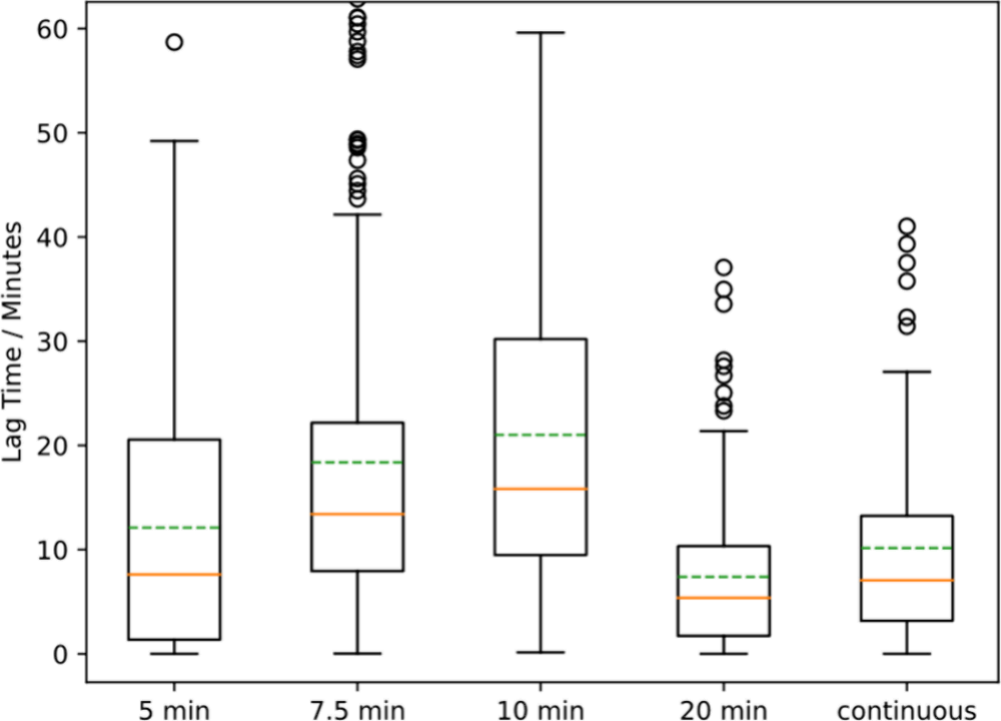
Lag times for *E. coli* as a function of exposure to 10 μg/mL PDADMAC. The horizontal axis indicates the time that 10 μg/mL PDADMAC was flowed over the cells before the flow was switched to water (switch flow experiment). Median Lag time decreases with more than 10 min of flow time. *p* = 10^–20^ for Mann–Whitney U test between the 10 and 20 min lag time data.

It is also of interest to know how the polymer surface density affects the time required for the cell to die (lag time). We know that the adsorption of PDADMAC increases with time, so we varied the time that PDADMAC flowed over the cells before the solvent was switched to water. As shown in Figure 9, the lag time decreases when the flow time was increased from 10 to 20 min, which is consistent with faster killing with more PDADMAC adsorbed to the surface.

### *E. coli, S. aureus,* and *P. aeruginosa* Differ in Their PDADMAC Response

**Figure 10:**
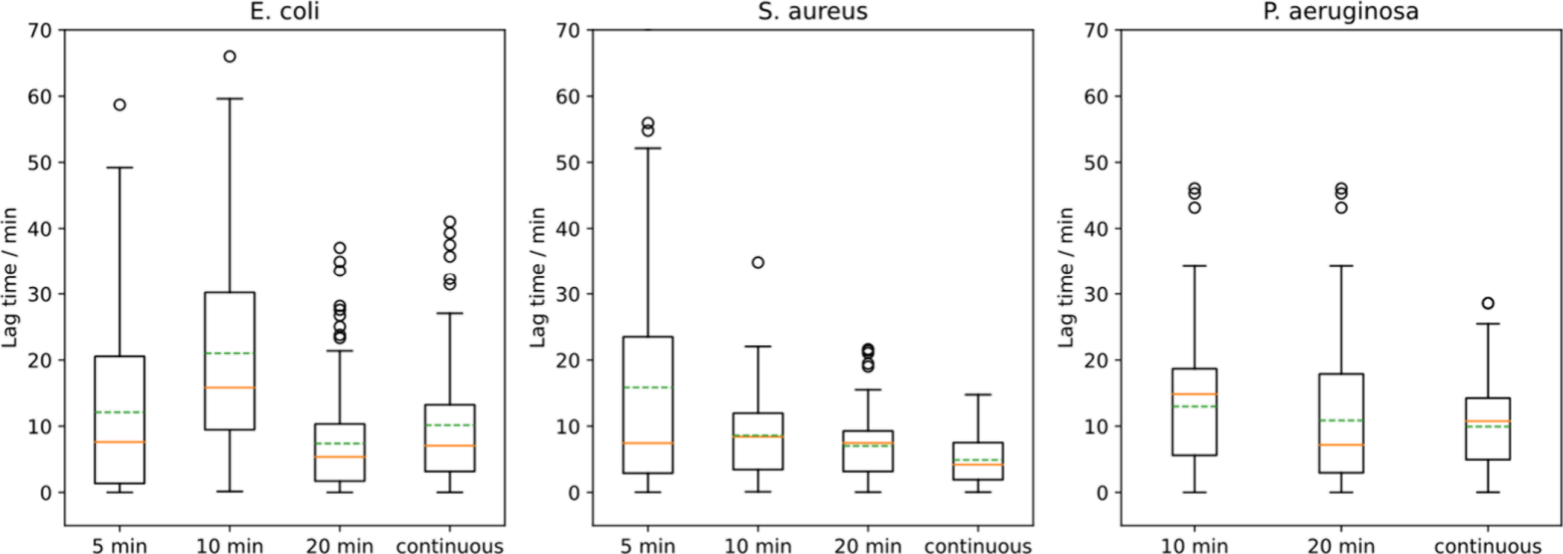
Lag time as a function of exposure of *E. coli*, *S. aureus*, and *P. aeruginosa* to 10 μg/mL PDADMAC. The horizontal axis indicates the time that 10 μg/mL PDADMAC was flowed over the cells before the flow was switched to water (switch flow experiment). *S. aureus* median lag time decreased with switch flow time similarly to *E. coli,* however, minimum median lag time for *S. aureus* (∼4 min) is lower than the minimum median lag time for *E. coli* (∼7 min), which is lower than the minimum median lag time for *P. aeruginosa* (∼15 min). *P* = 0.0003 and 0.04 for the Mann–Whitney U test between continuous flow for *S. aureus* and *E. coli,* and *P. aeruginosa* and *E.coli* lag time data, respectively, which we interpret as a significant difference between lag times.

**Figure 11:**
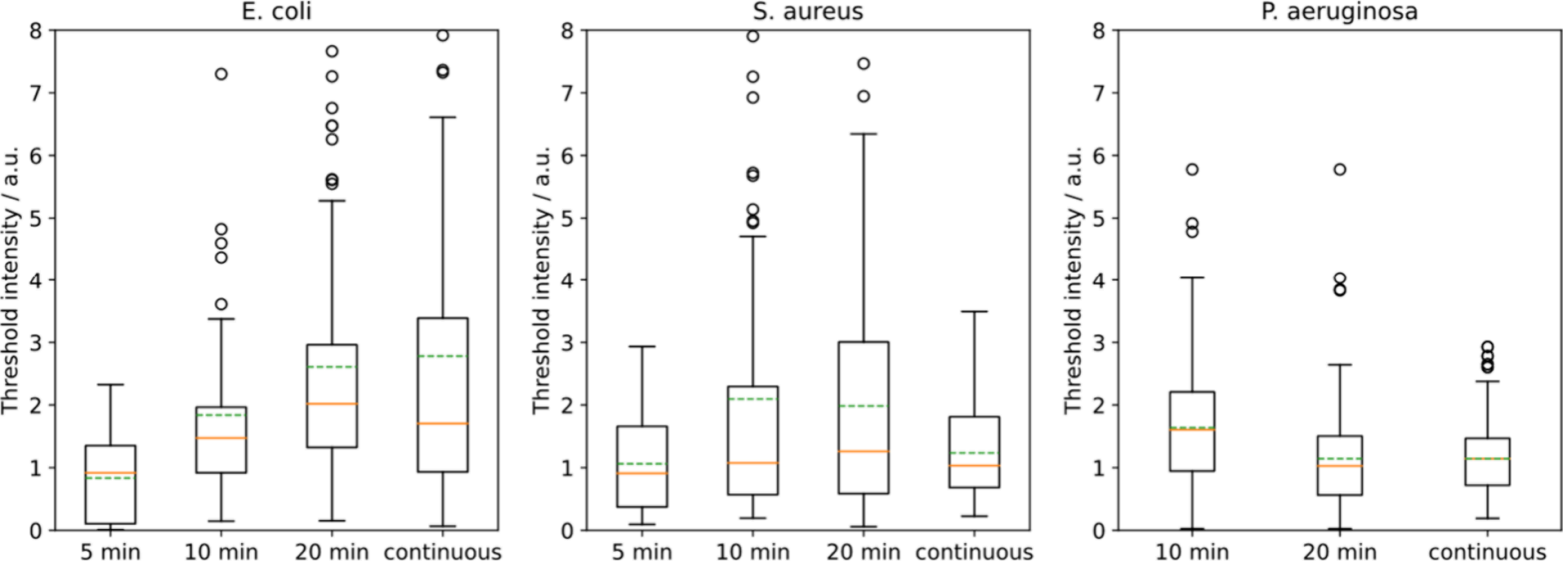
Threshold intensity as a function of exposure of *E. coli*, *S. aureus*, and *P. aeruginosa* to 10 μg/mL PDADMAC. The horizontal axis indicates the time that 10 μg/mL PDADMAC was flowed over the cells before the flow was switched to water (switch flow experiment). Median threshold intensity remains constant with switch flow time for *S. aureus* and *P. aeruginosa* as opposed to *E. coli,* where threshold intensity increased with switch flow time.

**Figure 12:**
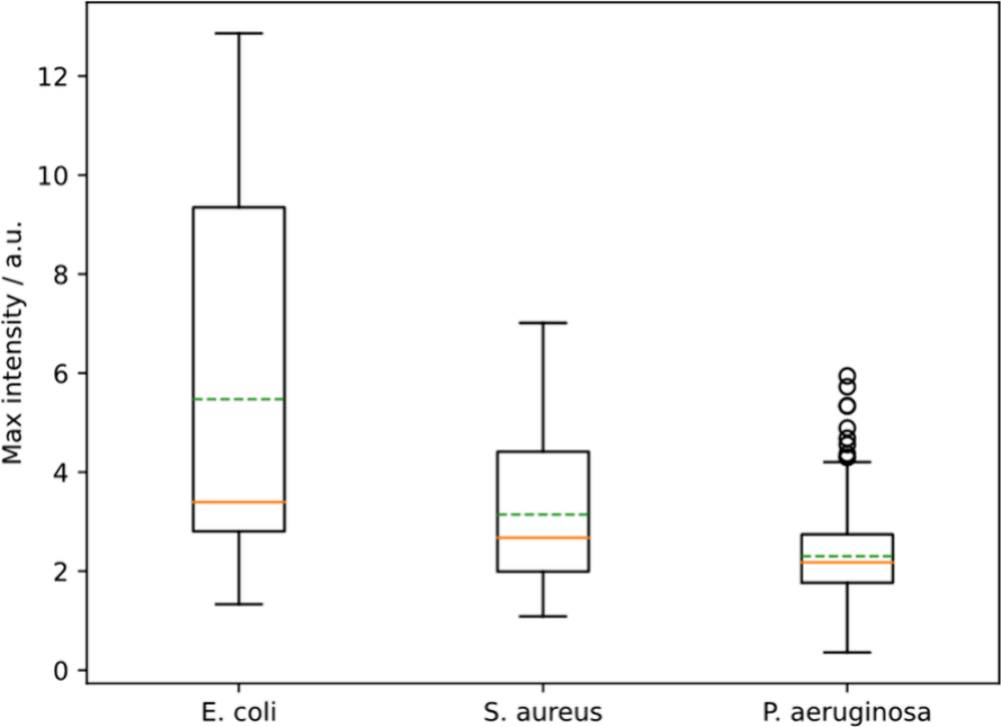
Maximum PDADMAC intensity for *E. coli*, *S. aureus*, and *P. aeruginosa*. Maximum intensity refers to the final equilibrium PDADMAC intensity for each cell when PDADMAC was flowed continuously over the cells. *E. coli* has a much larger capacity for PDADMAC adsorption than *S. aureus* (*p* = 7 × 10^–7^ for the Mann–Whitney U test), and *S. aureus* had a larger capacity for adsorption than *P.aeruginosa* (*p* = 5 × 10^–11^) which we interpret as significant differences in maximum adsorption. It is also of note that this trend in maximum intensity closely resembles the trend in zeta potential for each cell, with more negative zeta potentials leading to larger maximum intensities.^41,40^

It is of interest to know how different bacterial species respond to an antimicrobial. Here we examined differences between representative gram-positive (*S. aureus*) and gram-negative (*E. coli* & *P. aeruginosa*) species. Recall that gram-positive cells have only a single cell membrane and a thicker peptidoglycan layer so the initial adsorption of PDADMAC occurs onto different structures for the two cell types.^39^ There is a lag-time for all three species (Figure 10); for both cell wall types, a period of minutes passes after PDADMAC adsorption and before the cells become permeable. However, for *S. aureus* the lag time is shorter than for *E. coli* and *P. aeruginosa*. The longer time for the gram-negative bacteria may be due to the presence of an outer membrane, which PDADMAC must permeabilize before it can permeabilize the inner membrane. An important commonality between the three species is that there is a considerable diversity of response. This is seen both for the lag time and the threshold intensity (Figure 11). For *S. aureus* and *P. aeruginosa*, the threshold intensity remains constant with an increasing switch flow time, which is in contrast to the behavior observed for *E. coli* (Figure 7). A likely explanation for *S. aureus* this is that lag time is shorter so that there is less time for polymer to adsorb before the cell is observed to die, whereas for *P. aeruginosa*, it can be seen in Figure 12 that the maximum adsorption is much lower than *S. aureus* and *E. coli*, meaning that *P. aeruginosa* likely saturated much earlier, and its threshold would not change with increasing flow time.

The maximum adsorption of polymer decreases in the order *E. coli* then *S. aureus*, then *P. aeruginosa* (5.5 vs 3 vs 2.3, see Figure 12). All three bacteria have a negative zeta potential, and literature values of the magnitude of zeta potential, which is related to the surface charge, follow the same order as maximum adsorption.^40,41^ Adsorption of PDADMAC is primarily driven by electrostatic interactions and, so, will be curtailed when adsorption of the polymer causes the bacterium to have the same sign of charge as the polymer in solution. This will require less PDADMAC for the lower potential species, explaining the lower observed adsorption.

### Cells that Survive Exposure to PDADMAC Have a Lower Density of Adsorbed PDADMAC

We expected that surviving cells would have a lower density of adsorbed PDADMAC, but this was difficult to determine because so few cells survive, less than 1% in 10 min in 10 μg/mL PDADMAC according to CFU measurements. The issue was further complicated by the fact that live cells were identified by the absence of the “Dead Stain” i.e., by the absence of DNA staining. Stray light, interference patterns, and contaminating particles all do not stain for DNA, so the fraction of false positives is high when computer recognition of only a few cells. Therefore, each live cell was manually identified.

**Figure 13:**
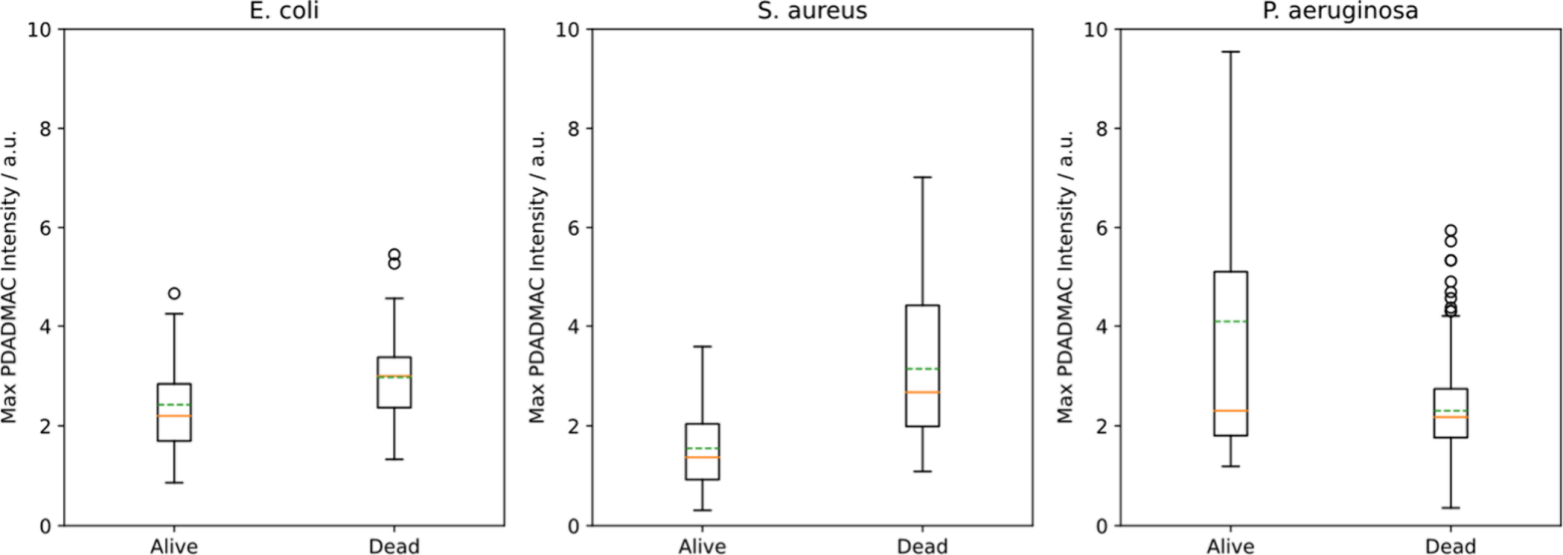
Comparison of PDADMAC adsorption on cells that live to those that die in 10 μg/mL PDADMAC. The adsorption was determined from the intensity of Cy3 dye on the polymer chain and is the maximum adsorption during the period rather than the threshold intensity because the threshold is not defined for a cell that does not die. Cells that survive typically have less adsorbed PDADMAC (*p* = 0.03 for *E. coli*, *p* = 5 × 10^–5^ for *S. aureus,* Mann–Whitney U test) but there is an overlap in the distributions. Additionally, for *P. aeruginosa*, there was no difference between surviving and killed cells (*p* = 0.125). Zero represents the background intensity. Data for 17, 15, and 23 surviving cells and 66, 75, and 371 dead cells for *E. coli*, *S. aureus*, and *P. aeruginosa*, respectively. Typically, the kill rate, measured by membrane permeability, was about 95%. This is much lower than the “kill” measured by CFU (∼99%). This may be because some of the loss of CFU may be cells that are not dead, but simply unable to reproduce (bacteriostatic).

Few live cells were found for continuous flow experiments, a total of about 10 per experiment. For these survivors, the median intensity was lower than that of the dead cells for *E. coli* and *S. aureus*, demonstrating that live cells have less adsorbed PDADMAC, as expected (*p* = 0.03) (Figure 13). This was not the case for *P. aeruginosa*. To obtain improved statistics, we also examined survivors of the switch flow experiments, of which there were about 50 cells in total in all of our experiments. These cells were exposed to PDADMAC solution for 7.5, 10, or 20 min and then rinsed with water for a further ∼ 60 min. Because PDADMAC did not rinse off, the total exposure to adsorbed PDADMAC was ∼70 min. These surviving cells also have less PDADMAC than the dead cells (*p* = 0.01) (Figure 13). Surprisingly, a few outliers had as much as 1.5–2 × as much as the median adsorption on cells that died. It is not clear how these cells could survive with that loading.

### The Cause of Persistence to PDADMAC

Our results show that harder-to-kill cells are typically those with low levels of PDADMAC adsorption. We considered several hypotheses for why the adsorption is low. One is that those cells have a low magnitude of the negative zeta potential. Generally, *E. coli* has a negative zeta potential, and the polymer is cationic, and the consequent electrostatic attraction should enhance adsorption of a cationic polymer. It is possible that there is a spectrum of zeta potentials, but it is difficult to measure both the zeta potential and the adsorption on the same cell, so we cannot test this hypothesis with the current technique.

To characterize resistant cells, we attempted to isolate a batch of resistant cells from the distribution that was present in all of our experiments. To do this, we exposed suspended cells to PDADMAC to kill susceptible cells and then incubated the survivors to produce a pool of resistant cells. We repeated this in a series of five rounds of exposure and incubation. The resultant cells experienced the same susceptibility to PDADMAC as the original cells (Figure S14), which suggests that the observed persistence is not inherited but is phenotypical. We conclude that during the incubation time (about 2 days or 144 generations assuming division every 20 min), with exponential growth of the population, the *distribution* of persistence to PDADMAC returns to the starting value. The distribution of persistence is a property of the entire population and somehow instructions for the distribution are communicated through the population. This phenomenon where a resistant subpopulation of monoclonal cells spontaneously develops is called heteroresistance.^26,42,25^

An alternative hypothesis is that persistence naturally occurs in certain parts of the life cycle. This would explain why some cells are resistant, yet not all their progeny are resistant. *E. coli* cells go through a life cycle where they grow and periodically divide. After 144 generations, there should be a distribution of cells in different stages of the life cycle. The cell membrane is about twice as large prior to division as afterward, and it is possible that the larger membrane has a different PDADMAC absorption capacity or other susceptibility compared with the smaller membrane. To test this hypothesis, we measured the lag time as a function of cell eccentricity (length to width ratio); cell eccentricity was used as an indicator of timing in the cell cycle. A plot of lag time vs eccentricity (Figure S15) makes it clear that we can easily resolve a large range of eccentricities, but there is no obvious correlation between eccentricity and lag time (*R* = −0.01). We conclude that the cell life stage was not an important determinant of persistence in cells that are not actively growing. In contrast to most antibiotics that only kill when cells are actively dividing,^43^ PDADMAC is able to kill when the cells are not dividing.

### Comparison to Prior Work

Antimicrobial peptides are typically highly cationic and therefore have a common feature with synthetic cationic polymers, although the application is different because the peptides are often active under physiological conditions, including at high ionic strength and often under conditions where cells are actively growing, whereas the synthetic polymers are used outside the body, usually at low ionic strength without a fuel source. Previous work by Sochacki et al. studied the effect of an antimicrobial peptide on growing *E. coli* cells.^17^ They found that the peptide saturated the cell surface within 1 min. They reported that cell growth was halted by translocation of the antimicrobial across the periplasmic space, which occurred about 5–25 min after initial adsorption. In their experiments, the peptide signal grew in steps over about 25 min and permeation of the cell wall began toward the end of this period. We found that PDADMAC adsorption increased continuously with the functional form of a logistic function, consistent with adsorption being limited by surface packing, and that permeation occurred more quickly, typically about 5–10 min after the beginning of adsorption. For actively growing cells, Sochacki et al. found that cells that are septating (commencing division) are more susceptible to antimicrobials, whereas we did not find a correlation between cell length (an indicator of timing of the cell cycle) and susceptibility for cells that are not actively growing. In contrast to prior results, PDADMAC does not appear to selectively adsorb to the junction of dividing cells or to have particular efficacy against cells that are long and therefore close to cell division. Work by Matthew and Nagaraj showed that some antimicrobial (cationic) peptides damage the inner membrane and some do not.^27^ Here we find that PDADMAC quickly makes the cell wall permeable for both gram-positive and gram-negative cells; thus, clearly the mechanism of action depends on some combination of the cationic structure and the test conditions.

### Comparison to MIC and MBC Measurements

MIC and MBC assess the minimum antimicrobial concentration to achieve inhibition of cell growth or to kill a particular fraction of cells at a particular time and therefore are population properties. In contrast, the metrics developed here, such as the amount of adsorption or the lag time, are properties, of individual cells and therefore are quite distinct from MIC and MBC. The individual properties, can be used to obtain the population properties, but not the reverse. For example, the average kill for a particular antimicrobial concentration in flow cell experiments can be compared to the MIC. However, such averages obscure the very great variation that occurs across the population. A MIC measurement does not reveal that the surviving cells have a different response: they are different phenotypes, rather than just the lucky survivors. An additional consideration is that we find that there is not really a concentration that kills, but rather a particular level of adsorption that needs to be achieved. That adsorption can be achieved in different ways, for example, a higher concentration at a shorter time or a lower concentration for a longer time.

## Conclusions

Bacteria are killed by PDADMAC once they are coated in a sufficiently high density of polymer but only after the passage of a characteristic lag period. So, there are two important time periods: the time to adsorb and the time to die. Both the lag time and the density of the polymer depend on the solution concentration. However, the amount of adsorbed polymer required to kill cells is independent of the solution concentration. The density of PDADMAC adsorption varies greatly among cells, even for a monoclonal population bathed in a common concentration of PDADMAC. A monomeric cation shows a similar diversity of adsorption. We conclude that variation in the charge of individual cells is the cause of the variability in the adsorbed density. The time-course of polymer adsorption fits well to a crowding model, and fitting allows a quantitative summary of the time course of adsorption. The cell life-cycle stage, as indicated by the cell length, does not affect the susceptibility of cells to PDADMAC. A similar density of polymer kills *E. coli*, *S. aureus*, and *P. aeruginosa*, but *S. aureus* dies faster and has a smaller maximum polymer density than *E. coli*, whereas *P. aeruginosa* dies the slowest, with the smallest maximum polymer density. Surviving cells generally had a smaller polymer density than killed cells, suggesting that a source of PDADMAC persistence is due to or at least correlated with a low capacity for PDADMAC adsorption. Replication of surviving cells did not lead to a population of cells that are resistant to PDADMAC, indicating that adsorption capacity and subsequent persistence are probably phenotypical in nature.

We showed that a threshold adsorbed polymer density was required to kill cells but also demonstrated that this was not a sufficient condition to kill cells. A few hardy cells had high adsorption but did not die, and it is unclear exactly why that is the case. There is some data suggesting that there is a threshold zeta potential that must be reached to kill cells,^9^ which correlates with our results of cell death requiring a threshold amount of adsorption. However, the two concepts are not the same, because different cells can differ in the initial zeta potential. The latter seems likely given the huge variation in adsorption that we observe from cell to cell.

Simultaneous time-resolved analysis of every cell in a population under the same conditions is useful for understanding the dispersion of cell behavior, lag times, and other variables that are properties of individual cells. Measurement of many members of a population allowed us to observe the wide range of adsorptions that occur and to observe persistent cells that inherently have a lower capacity for adsorption or are unaffected by high adsorption. An advantage of fluorescence imaging compared with flow cytometry is that microscopy can measure properties of each cell in the population over time. Few properties that we measured are summarized well simply by an average, and most properties were not normally distributed. By comparison, standard MIC measurements usually measure the concentration for a particular fraction of cells to die in a given time, which contains a very reduced data set. For example, one might conceivably think of the survivors in an MIC experiment to be statistical survivors (the lucky ones), whereas we find that cells with different survival characteristics have different properties.

The variables described using this analysis (threshold intensity and lag time in particular) provide a useful tool in analyzing antimicrobial efficacy as well as providing insight into the mechanisms of antimicrobials. For instance, in the design of antimicrobials, fast action is desirable. We found a time lag between polymer adsorption and cell membrane permeation, which is much longer than transport times and therefore is the rate limiting step. It is difficult to identify the rate limiting step in traditional plate count (colony forming unit) experiments because the assay is made at the end point so separate events are not directly observable. Additionally, we find that the threshold adsorption (the density of antimicrobial that is needed to kill cells) rather than the concentration in solution is correlated with cell permeation. Our results suggest that an effective antimicrobial is one that has a very small threshold adsorption. Mechanistically, the presence of a threshold intensity in general suggests that not only the presence of adsorption but also the amount of adsorption is relevant in the mechanism. From a practical viewpoint, we have identified that some cells survive even with a very heavy load of antimicrobials that easily kills other cells. One way to improve the efficacy would be to include a second antimicrobial that specifically targets these cells.

## Supporting Information Available

Derivation of eq 1: the half-logistic equation, photograph and schematic of the flow cell, killing of *E. coli* in suspension, frequency distribution of time of death of *E. coli* cells in water, range of Rhodamine 6G adsorption, emission of quantum dots for calibration (PDF)
