# Supplementary material for: Time-Resolved Killing of Individual Bacterial Cells by a Polycationic Antimicrobial Polymer
Source: ACS Biomater Sci Eng. 2024 Mar 29;10(5):3029–40. doi: 10.1021/acsbiomaterials.4c00263 (PMC11094676; doi:10.1021/acsbiomaterials.4c00263)
Supplement: Supplementary file 1 — ab4c00263_si_001.pdf [file ab4c00263_si_001.pdf]

Supporting Information

Time-Resolved Killing of Individual Bacterial Cells by a Polycationic Antimicrobial Polymer

Zachary Benmamoun[1], Prem Chandar[2], Joe Jankolovits[2], William A Ducker[1]

[1.] Department of Chemical Engineering, Virginia Tech, Blacksburg, VA, 24060, USA

[2.] Unilever

Derivation of Equation 1: the Half-Logistic Equation

To simplify comparison of adsorption–time data among cells within a population, we sought an equation that would fit well to our data and allow us to reduce this time-course to only a few parameters. We chose the half-logistic equation (Equation 1) and justify its use by deriving it here with stated assumptions. We assume that the fluorescence emission is proportional to the number of polymer molecules. We further assume that the attachment of polymer chains depends on the collision between a molecule in solution and a vacant site on the solid. Because constant flow of polymer replenishes depleted polymer, we assume a constant polymer concentration in solution, and the process is therefore pseudo first-order. Because of electrostatic interactions, one would expect that the number of adsorption sites would depend on the polymer conformation, which in turn depends on the solution conditions such as salt and polymer concentration. For simplicity, here we assume that the number of sites is constant over the time-course for any particular solution condition. We define the following:

Bulk polymer concentration $\equiv c$

Fraction of occupied sites $\equiv \theta = \frac{N}{N_0}$, where N is the adsorbed number and N_0 is the number of sites, which is a function of solution conditions.

Fraction of vacant sites = $1 - \theta$.

For a second order process, $\frac{dN}{dt} = k'c(1-\theta)$,

where k' is a second order rate constant. For fixed polymer concentration, $k \equiv k'cN_0$ is constant, so

$$\frac{d\theta}{dt} = k(1-\theta).$$

For ease of solution, we make the equation homogeneous by substitution of the fraction of unoccupied sites, $\theta' \equiv 1 - \theta$:

$$-\frac{d\theta'}{dt} = k'\theta'$$

We set t_0 to be the time when the polymer first arrives near the cell, and at that time all sites are unoccupied. For this initial condition, the solution is:

$$\theta' = \exp\left(-k(t-t_0)\right)$$

i.e., equation 1:

$$N = N_0\left[1 - \exp\left(-k(t-t_0)\right)\right]$$

Effect of Variation in Degree of Labelling on the Intensity to Adsorption Transform.

We treat the intensity of fluorescence emission as if it is proportional to the polymer coverage. There is on average 1 fluorophore per 196 monomer units, but the variation is unknown. If there are very few fluorophores on a bacterium, then we would expect variation in the degree of labelling among polymers to introduce random errors into our reported values of adsorption. The key to overcoming this problem is to have a large average number of fluorophores per bacterium so that such fluctuations average out. The following approximate calculation estimates the number of fluorophores per bacterium.

Assumptions:
E. Coli is a cylinder of length 2000 nm and diameter 1000 nm.
The area per monomer is 1 nm^2.
There is a monolayer of monomers on the bacterium.

The number of fluorophores per bacterium is then about 4×10^4.

This is a large number, so we expect that there will only be a small fluctuation in the number of fluorophores per bacteria for a given polymer coverage.

The weakest assumption is monolayer coverage. But even for 2% coverage, there will still be on average one thousand fluorophores per bacterium. If the polymer straddles three dimensions, there may be a larger number of polymer molecules and therefore fluorophores on the bacterium.

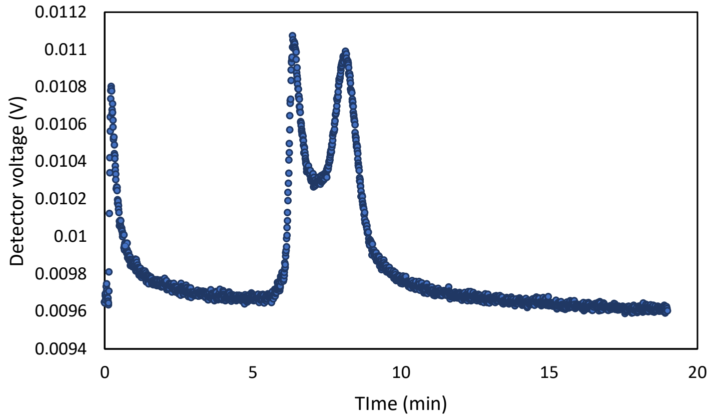

	Peak 1	Peak 2
Relative amount	45%	55%
M_n/gmol^{-1}	3.0×10^5	4.6×10^5
M_w/gmol^{-1}	1.1×10^6	5.6×10^5
M_w/ M_n	3.7	1.2

Figure S1. Light scattering as a function of time for fluorescently-tagged PDADMAC exiting size exclusion chromatography (SEC) column. There is a bimodal distribution of polymer with two fractions of similar molecular mass. Results from a Zimm model in the table below. These calculations use a PDADMAC dn/dc of 0.189.[1]

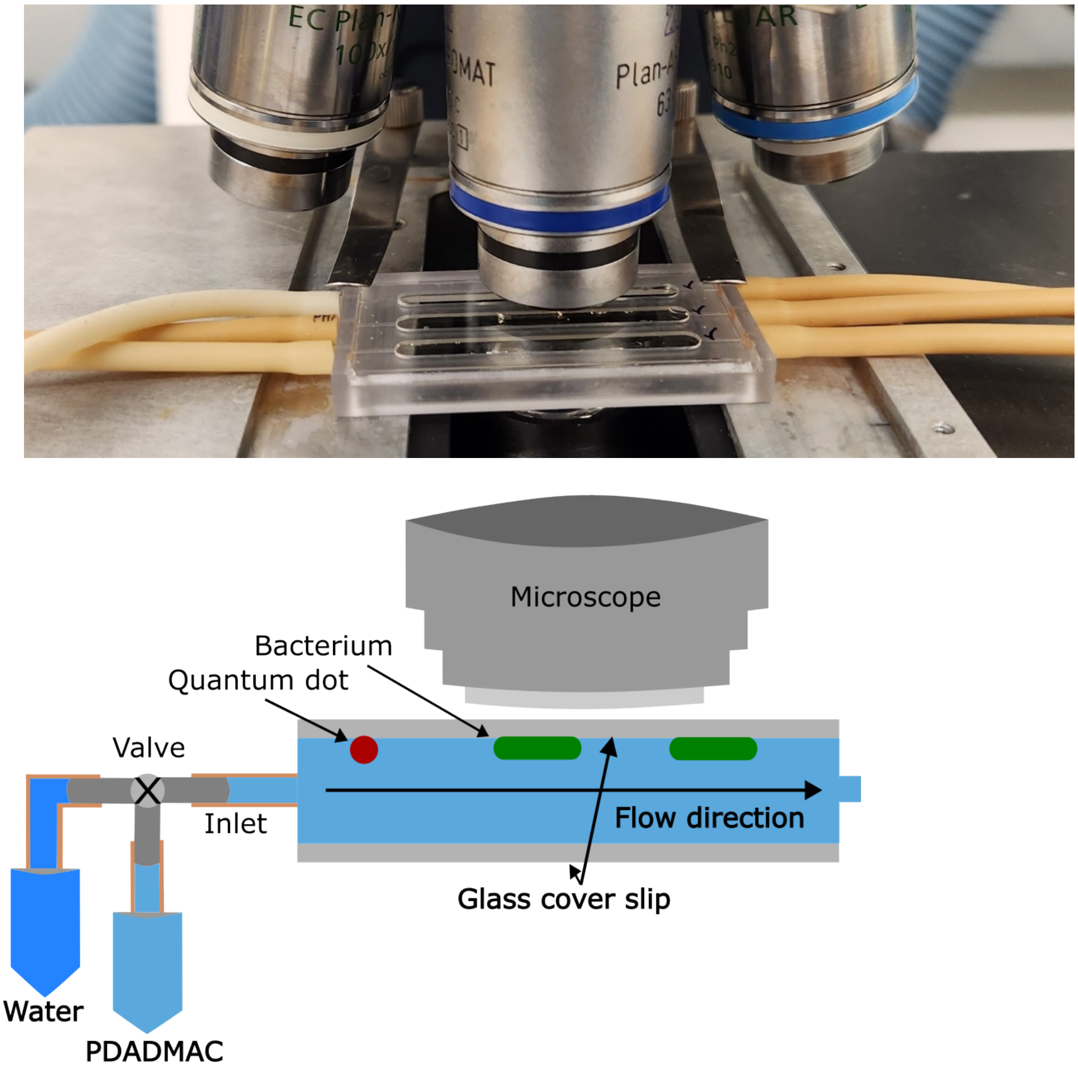

Figure S2. (A) Photograph of the flow cell. The flow cell was constructed from polycarbonate with the top and bottom consisting of glass coverslips adhered to the flow cell using polydimethylsiloxane (PDMS). Between experiments, the flow cell was sterilized using 70% ethanol, then the cover slips were removed and replaced with new cover slips before the next use. (B) Schematic of flow cell.

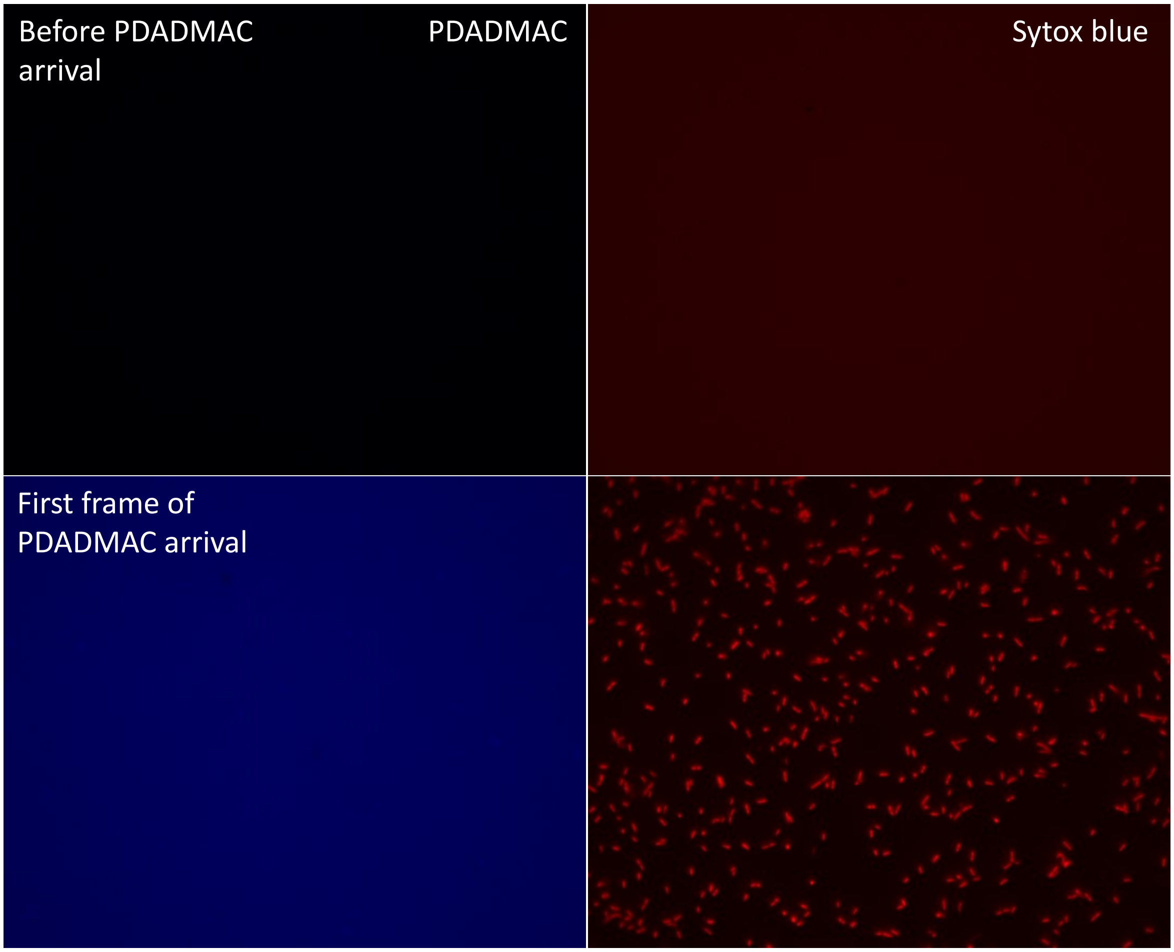

Figure S3. Test of diffusion time of Sytox Blue within the cell cytoplasm. There are four transport times for Sytox blue: (1) transport to the region of the cell, (2) transport (diffusion) from bulk solution near the cell to the surface of the cell, and (3) transport through the cell wall and (4) diffusion within the cell. Transport to the cell was less than two minutes, as evidenced by fluorescence of the cy3-PDADMAC adsorbed to glass around the cell and faint fluorescence of Sytox blue when it binds to extracellular DNA throughout the flow cell. About two minutes later, the cell fluoresces from cy3-PDADMAC, indicating adsorption. The time for these two is not relevant to the lag time (the time to die after adsorption) because the zero for the lag time is defined to be when the PDADAMAC first adsorbs. The time to die includes events at the membrane as well as diffusion through the cytoplasm. The diffusion time through the cytoplasm is expected to be short because it occurs over a very small distance (<0.5 µm). Diffusion time scales with the square of the distance; for example, a simple ion in water diffuses over 0.5 µm in 10^{-5} s. The inside of a cell is more gel-like but diffusion times within gels are well known. For example, the small polymer, in a polyacrylamide gel diffuses over 0.5 µm in less than one second ("Diffusion in polyacrylamide gels" Brown and Johnsen, *Polymer*, 1981). The images above are consistent with this expectation. We adsorbed cells in the flow cell, killed them with ethanol and then exposed them to Sytox blue and PDADMAC and found that the die for fluorescent to appear was only a few seconds, i.e., less than a single frame (30 s). Therefore the lag time is predominantly the time for the PDADMAC to render the cell wall permeable, rather than the diffusion time within the cytoplasm.

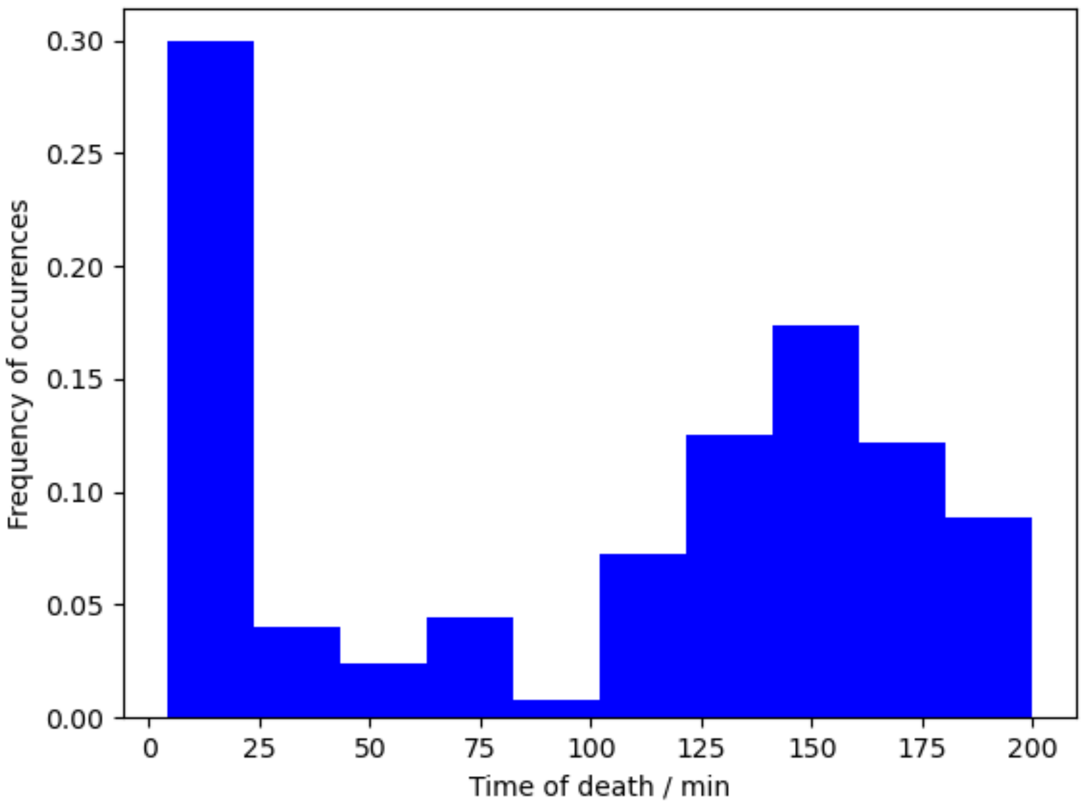

Figure S4. Frequency distribution of time of death of *E. coli* cells in water. Death measured with Sytox blue. Most cells start to die about 100–200 min after immersion in water. Some cells are dead at the beginning of the experiment and appear in the first bin. There is a finite time between when flow begins and when Sytox reaches the cells, and these initially dead cells are registered in the first minute, not in a “zero time” bin.

```python
#Calls fluorescence microscopy movies
img_cy3 = io.imread("10ppmPDADMAC_0.15MNaCl_cy3.tif") #PDADMAC channel
img_sytox = io.imread("10ppmPDADMAC_0.15MNaCl_sytox.tif") #Sytox channel
img_phase = io.imread("10ppmPDADMAC_0.15MNaCl_phase.tif") #Phase contrast channel

#Thresholds data
thresh_cy3 = threshold_otsu(img_cy3) #thresholds data using otsu method
thresh_sytox = threshold_otsu(img_sytox)
thresh_phase = threshold_otsu(img_phase)

#Creates binary images by removing background
binary_cy3 = np.ndarray(shape=img_cy3.shape) #Creates array same size as movie
binary_cy3[img_cy3<thresh_cy3]=0 #Sets pixel values below threshold to 0 brightness (removes background)
binary_cy3[img_cy3>thresh_cy3]=img_cy3[img_cy3>thresh_cy3] #Adds pixel values above threshold
```

Figure S5. Excerpt of Python code for thresholding fluorescence microscopy time lapse photographs.

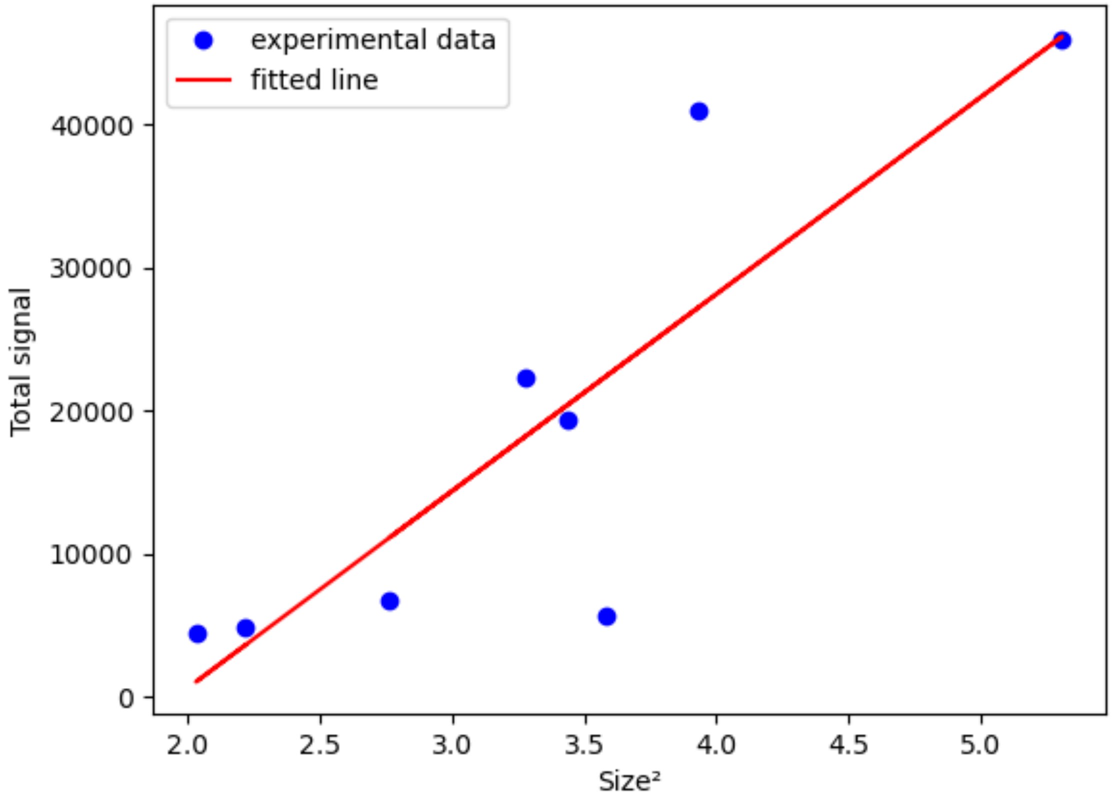

Figure S6. (A) Data for normalization of fluorescence intensity showing measured total signal from a single quantum dot (QD) as a function of area when no bacterial cells were present. The area was obtained from the square of the radius of gyration of a normal distribution to the fit and is in units of pixels. For example, a binary image covering 4 pixels would have a size squared of 4. The slope of the fit

is a measure of intensity emitted from the QDs and was used to calibrate the intensity in each bacterial experiment. R = 0.85, indicating a strong linear correlation. A minimum of 5 quantum dots was used for each bacterial experiment to account for variation amongst individual quantum dot emission.

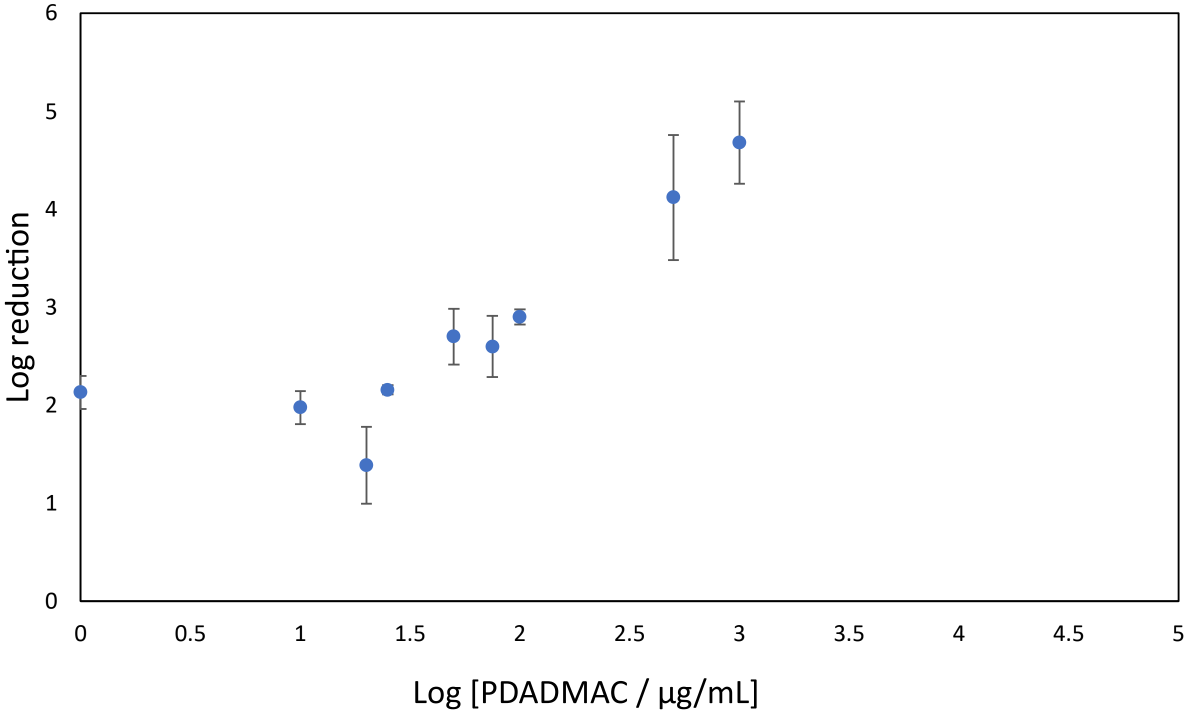

Figure S7. Reduction of *E. Coil* by PDADMAC in solution in 5 min as defined by:

$$\text{log Reduction} \equiv \text{mean}[\log_{10}(\text{CFU of suspension without antimicrobial})] - \text{mean}[\log_{10}(\text{CFU of suspension with antimicobial})]$$

Here the mean was taken over three replicates and CFU is a technique that counts cells that divide enough times to form a visible bacterial colony on agar.   Error bars show the 95% confidence interval for each condition. At 1 and 10 µg/mL, which are the concentrations used for the majority of the flow cell experiments, the reduction caused by PDADMAC is about 99%.

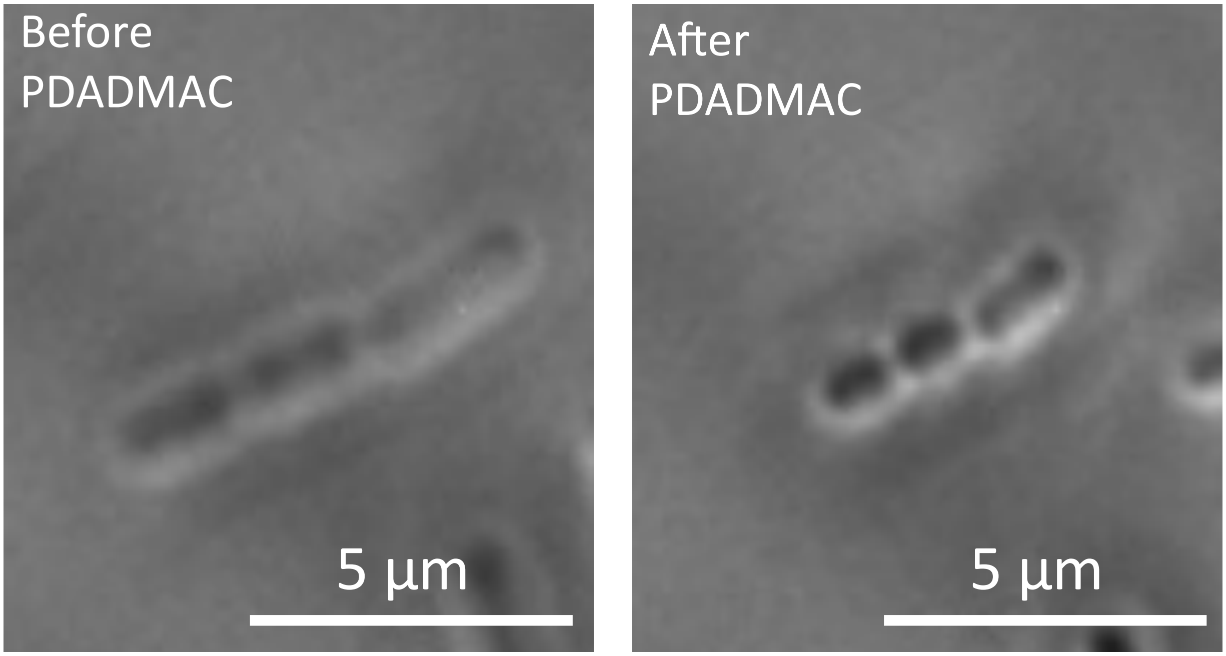

Figure S8. Phase contrast images of individual *P. aeruginosa* cell before (left image) and after (right image) 45 minutes of 10 ppm PDADMAC flow. No visible damage has been done to the cell other than the size decreasing. Cells do not lyse when exposed to PDADMAC flow.

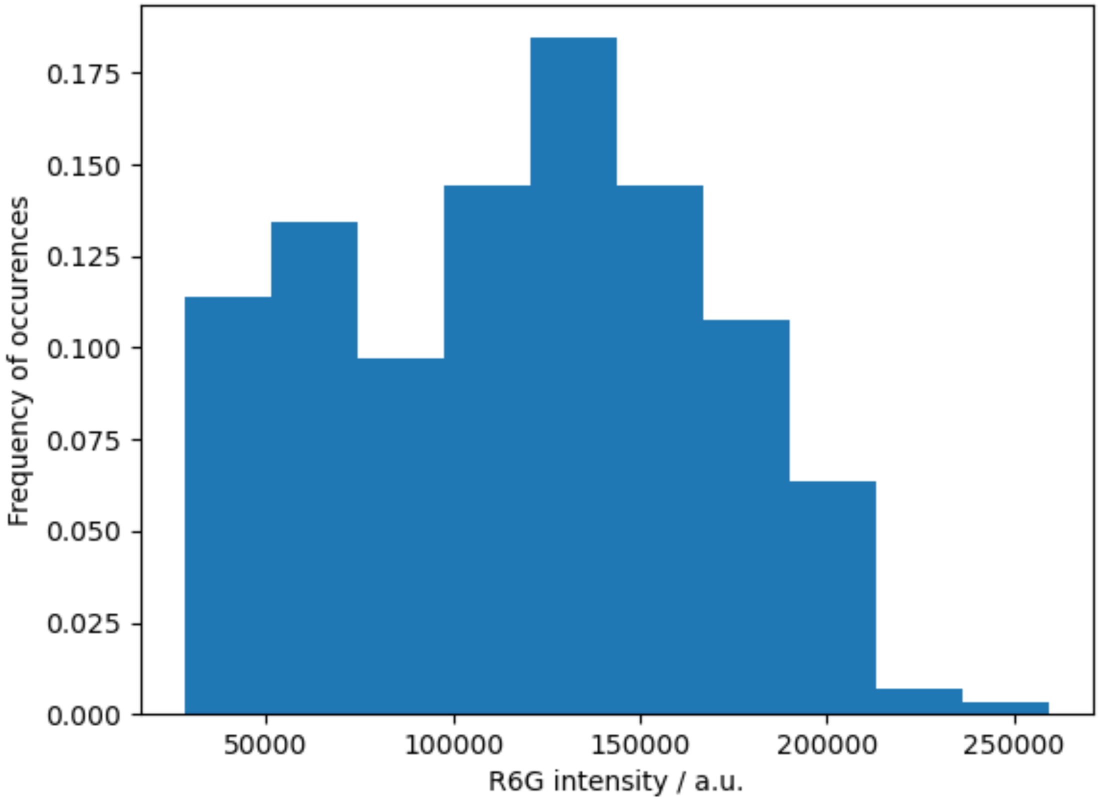

Figure S9. Range of Rhodamine 6G adsorption onto *E. Coli* in flow cell. Adsorption of this cationic molecule varies between cells, which is similar to the behavior of PDADMAC.

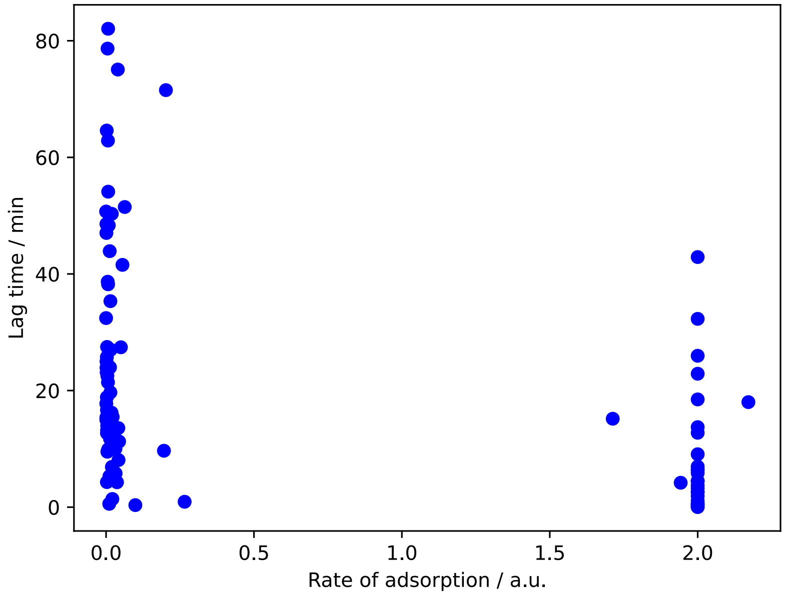

Figure S10. Rate of adsorption of PDADMAC onto *E. coli* cells vs lag time. Faster rates of adsorption correlate with smaller lag times.

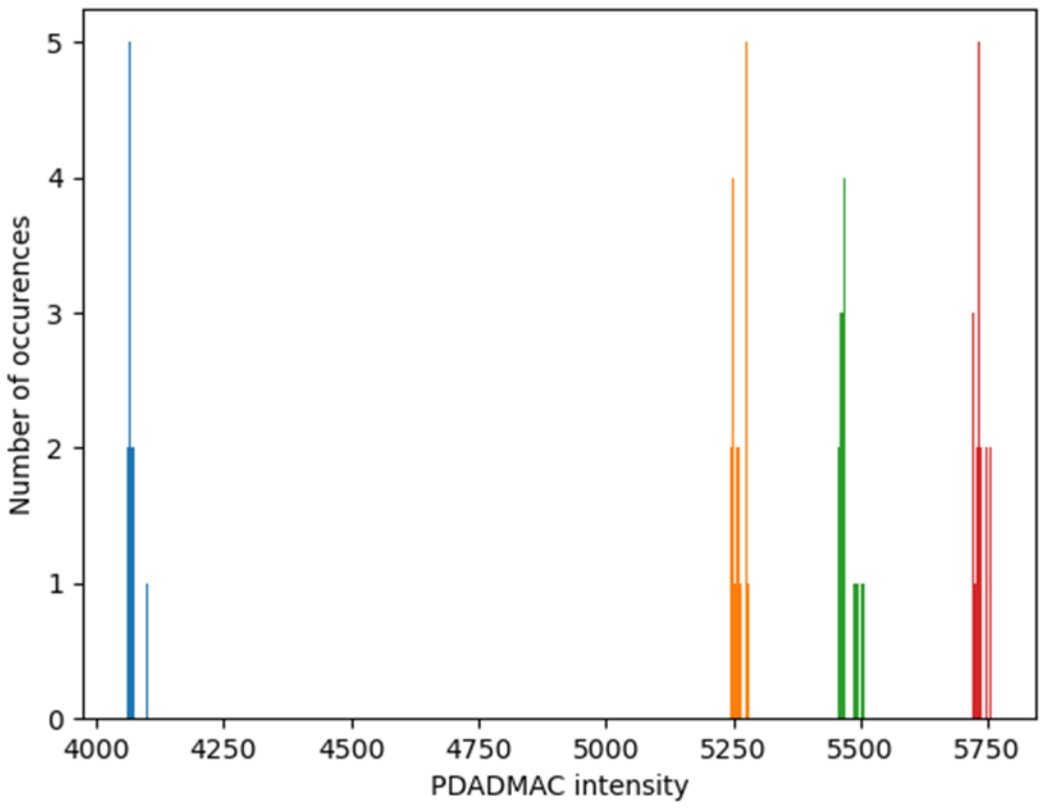

Figure S11. Fluorescence intensity of *E. coli* cells that have been exposed to 10 µg/mL Cy3-PDADMAC in suspension, mixed by vortexing and then photographed in a flow cell. Four replicated experiments are shown. We reach two conclusions from this experiment: (1) the heterogeneity in adsorption is a property of the cells, not the mixing in the flow cell, and (2) there is variation in mean intensity between replicates.

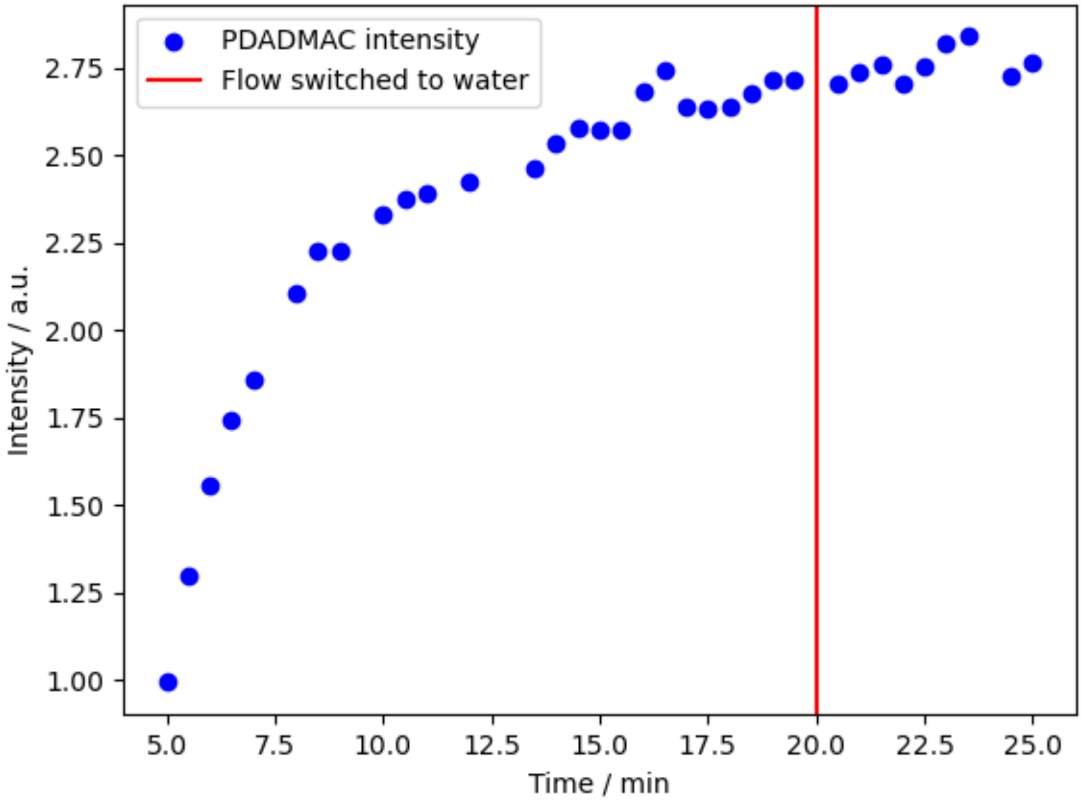

Figure S12. PDADMAC adsorption on a single cell during a switch-flow experiment at a constant PDADMAC concentration of 10 μg/mL. After 20 minutes, the flow of PDADMAC was switched to a flow of water, and the intensity did not drop. This indicates that adsorption was irreversible on this time scale.

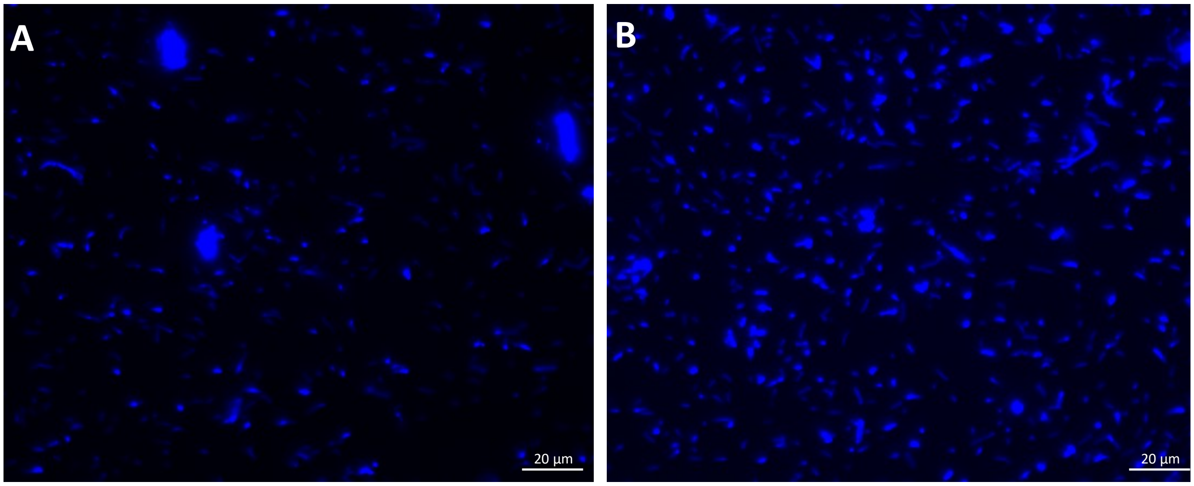

Figure S13. Time course of PDADMAC adsorption onto *E. coli* in switch flow experiments. A. 10 minutes of PDADMAC solution followed by 50 minutes of water. B. 20 minutes of PDADMAC flow, followed by 50 minutes of water. PDADMAC density increased with longer periods of PDADMAC flow.

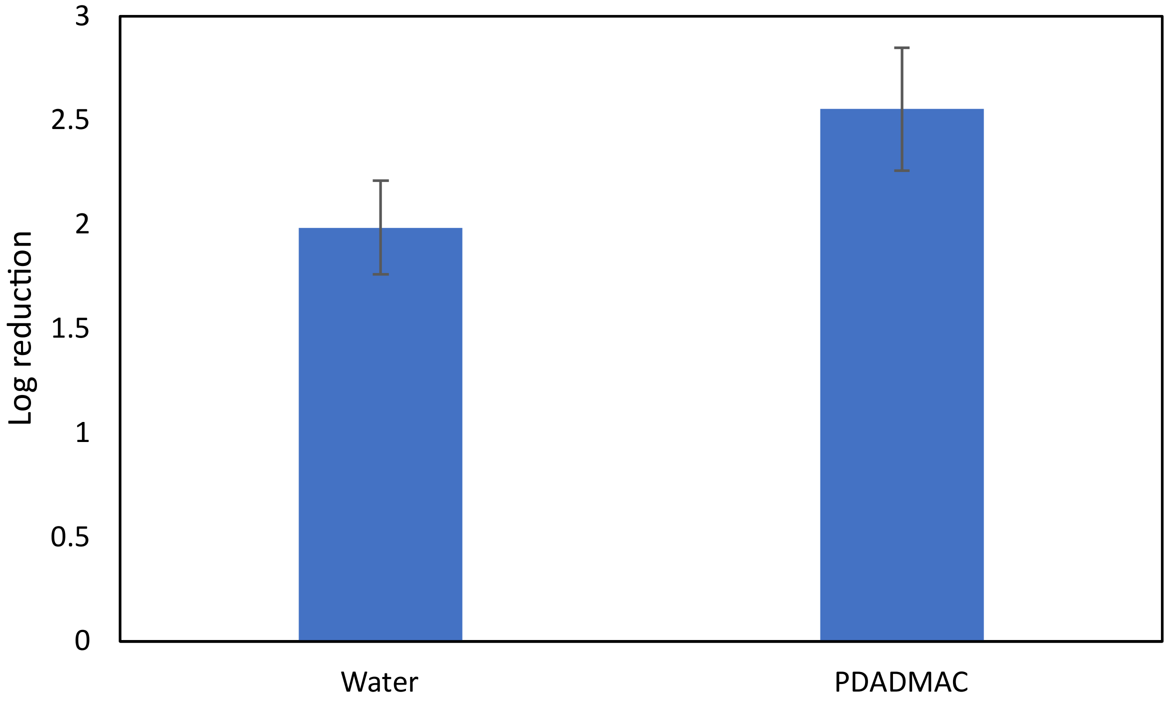

Figure S14. Effect of prior exposure of *E. Coli*. to PDADMAC on resistance to PDADMAC. *E. coli* was cultured in suspension of 10 µg/mL PDADMAC for 15 minutes, then plated on agar. Surviving colonies were picked, grown in TSB for 48 h, then exposed to 10 µg/mL PDADMAC for 15 minutes again. This was repeated a total of 5 times. A final CFU assay revealed similar log reduction of the cells for PDADMAC-passaged cells and water-passaged cells (2.5 vs 2.0, p = 0.65). 10 µg/mL PDADMAC causes a 2-log reduction of suspended cells in 5 minutes (Figure S4). We know from flow cell experiments that PDADMAC causes a loss of cell permeability, so PDADMAC kills the cells. This selective pressure of exposure to PDADMAC did not lead to an increase in the resistant proportion of the cell population. This suggests that, in this case, resistance is not a genetic trait of an individual cell but is a function of the population of cells; the distribution of resistance arises spontaneously after the population balance is perturbed by an external force.

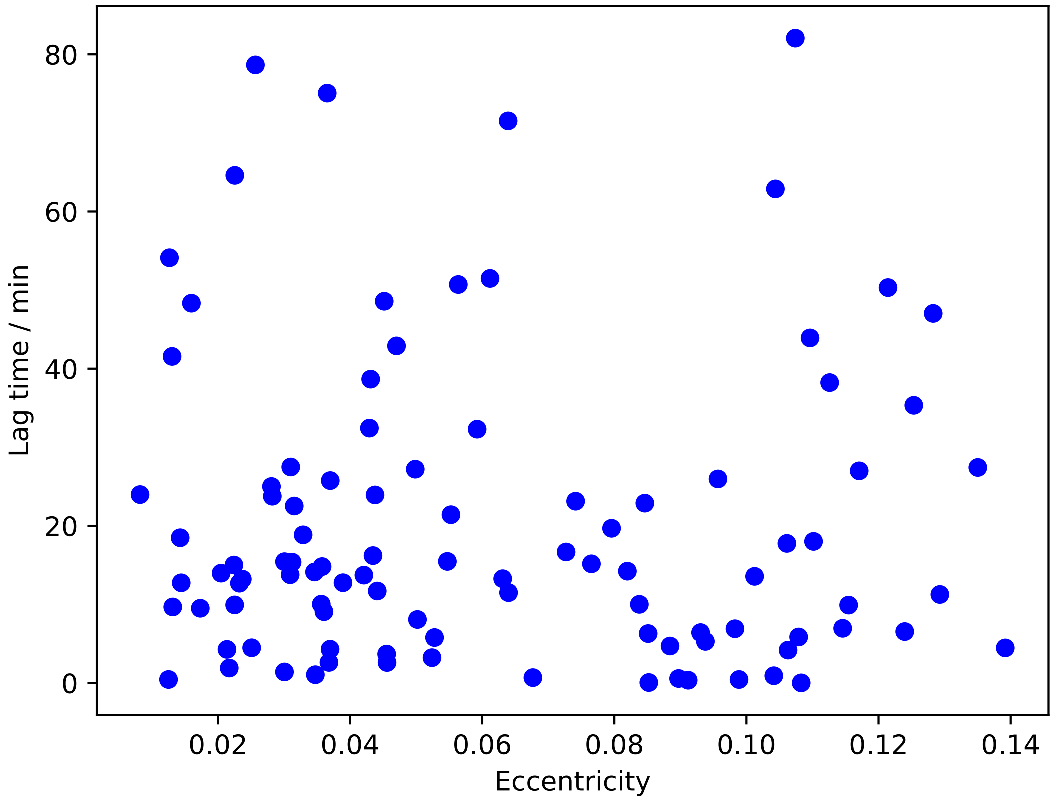

Figure S15. Lag time between adsorption and death as a function of cell eccentricity. R = -0.019. Eccentricity not correlated with lag time.

References

(1) Liu, X.; Haddou, M.; Grillo, I.; Mana, Z.; Chapel, J.-P.; Schatz, C. *Soft Matter* **2016**, *12* (44), 9030-9038.
